# Heavy weather events, water quality and gastroenteritis in Norway

**DOI:** 10.1016/j.onehlt.2021.100297

**Published:** 2021-08-04

**Authors:** Bernardo Guzman Herrador, Vidar Lund, Wenche Fonahn, Hege Hisdal, Hans Olav Hygen, Susanne Hyllestad, Zuzana Nordeng, Reidun Gangstø Skaland, Linda Selje Sunde, Line Vold, Richard White, Wai Kwok Wong, Karin Nygård

**Affiliations:** a Norwegian Institute of Public health, Postboks 222 Skøyen, 0213 Oslo, Norway; b Norwegian Meteorological Institute, Henrik Mohns Plass 1, 0371 Oslo, Norway; c Norwegian Water Resources and Energy Directorate, Postboks 5091, Majorstua, 0301 Oslo, Norway

**Keywords:** Climate change, Extreme weather, Gastroenteritis, Health implications, Norway, Water quality

## Abstract

Climate change will lead to more extreme weather events in Europe. In Norway, little is known about how this will affect drinking water quality and population's health due to waterborne diseases. The aim of our work was to generate new knowledge on the effect of extreme weather conditions and climate change on drinking water and waterborne disease. In this respect we studied the relationship between temperature, precipitation and runoff events, raw and treated water quality, and gastroenteritis consultations in Norway in 2006–2014 to anticipate the risk with changing climate conditions. The main findings are positive associations between extreme weather events and raw water quality, but only few with treated drinking water. Increase in maximum temperature was associated with an increase in risk of disease among all ages and 15–64 years olds for the whole year. Heavy rain and high runoff were associated with a decrease in risk of gastroenteritis for different age groups and time periods throughout the year. No evidence was found that increase in precipitation and runoff trigger increased gastroenteritis outbreaks. Large waterworks in Norway currently seem to manage extreme weather events in preventing waterborne disease. However, with more extreme weather in the future, this may change. Therefore, modelling future climate scenarios is necessary to assess the need for improved water treatment capacity in a future climate.

## Highlights

• Positive associations between extreme weather events and raw water quality.
• Increase in maximum temperature was associated with an increase in risk of disease.
• Heavy rain and high runoff were associated with a decrease in risk of gastroenteritis.
• Larger water works in Norway seem to cope with the extreme weather events in the current climate.

## 1 Introduction

Climate change is predicted to lead to more frequent extreme weather events in the European region [1]. In the Nordic region the detected temperature increase is larger than in central and southern Europe, and this is expected to continue. This region has also experienced an increase in annual average precipitation that is expected to continue as opposed to central and southern Europe, where no change or even a decrease can be expected. The rate of change towards the end of the century is highly dependent on the choice of emission scenario. With the high emission scenario (RCP8.5) the annual temperature in Norway may increase by 4.5 °C and the annual precipitation may rise by 18% by the end of this century. Scenarios that are more moderate show less response with for example a median temperature increase of 2.7 °C for RCP4.5. Heavy rainfall and subsequent pluvial floods will be more intense and frequent also in Norway, but again the degree of change will depend on the rate of climate gas emissions [2].

Evidence is accumulating on the links between extreme weather events and drinking water quality [[3], [4], [5]]. Studies have shown that concentrations of coliform bacteria, *E. coli*, enterococci, and enteroviruses increase in environmental samples following rainfall. During extreme rainfall and runoff events, other water quality indicators, such as the turbidity, pH, and nitrate values are also influenced. Heavy rainfalls can rapidly elevate the total organic carbon and particles in surface water affecting the transport of microorganisms [[3], [4], [5]]and increasing the risk and burden of waterborne disease. Surface water is particularly vulnerable to changes in temperature, heavy rainfall and floods. In general, Nordic countries get most of their drinking water from groundwater, but as 90% of Norwegian drinking water is obtained from surface water [5,6], the water supply could be particularly impacted in case of increased extreme precipitation and floods.

In Norway, little is known about how extreme weather events affect drinking water quality and health. Our aim was to enhance the current understanding of the association between extreme weather events, water quality and waterborne disease by using epidemiological, hydrological, and meteorological data. And, we have studied to what extent extreme weather events during the past years have influenced the quality of raw and treated drinking water, and the subsequent risk of waterborne disease.

## 2 Material and methods

### 2.1 Data sources

#### 2.1.1 Water quality data

We used the National waterworks registry (VREG) to select the 26 largest waterworks in Norway for the study (with a total of 37 water treatment plants): including 23 surface water and three groundwater works (Fig. A). VREG include all Norwegian waterworks supplying more than 50 persons. We choose the biggest waterworks, serving approx. 60% of the Norwegian population, because the smaller waterworks did not have sufficient data to be included in the statistical analysis for associations with climate parameters. Water treatment at the included waterworks, except for the three groundwater works, include at least coagulation, UV disinfection and chlorination. The groundwater works had protected aquifers and disinfection (in stand-by). Water quality data for raw water (source water) and treated water (clean/distributed) on the following parameters were collected: *E. coli*, intestinal enterococci, coliform bacteria, heterotrophic plate counts, turbidity, colour, pH and conductivity, for the period 2006–2014 (only available data when implementing the study). The indicator bacteria: *E. coli*, intestinal enterococci, coliform bacteria, and heterotrophic plate counts, are standard part of water quality surveillance in Norway following the Drinking Water Directive 98/83/EC [7].

We requested accredited water quality data from routine monitoring scheme, data from internal control and data from on-line raw and treated water monitoring from the selected waterworks.

#### 2.1.2 Gastroenteritis consultations

We extracted data on gastroenteritis consultations in Norway during the period 2006–2014 from the Norwegian Syndromic Surveillance System (NorSySS) [8] operated by the Norwegian Institute of Public Health (NIPH). NorSySS monitors how many infectious diseases are reported during consultations with general practitioners (GPs) and out-of-hours primary care facilitiesNorSySS contains data about the patient's age group, gender and the municipality of residence, the municipality where the consultation was performed, date and diagnosis code in the International Classification of Primary Care system (ICPC-2). Gastrointestinal infections are a collective term for the diagnosis codes (ICPC-2) D11-Diarrhoea, D70-Gastrointestinal infection and D73-Gastroenteritis presumed infection. The Fig. B shows somewhat increased number of consultations with these diagnostic codes since 2006, but remaining stable over recent years, with over 200,000 consultations per year. [8]. NorSySS does not provide the exact number of infected people since some people will contact their GP several times with the same diagnosis and others may not consult their GP at all. NorSySS uses Quasi-Poisson Regression and the Farrington method to estimate the expected number of cases during a non-outbreak period. We then applied this algorithm to each municipality using weekly data and defined an outbreak as a week when the observed value was higher than two standard deviations from the expected non-outbreak baseline.

#### 2.1.3 Meteorological and hydrological data

The Norwegian Meteorological Institute (MET) and the Norwegian Water Resources and Energy Directorate (NVE) provided gridded data for runoff (m^3^/1000m^2^), accumulated daily precipitation, daily mean temperature, and observed daily maximum temperature. Precipitation at daily mean air temperatures above 0.5 °C was defined as rain. Precipitation is measured at around 400 locations in Norway, with slight technique variations. At older stations, meteorologists measure manually, with every monitoring representing the past 24 h. At newer stations, measurements are automatic and conducted every hour based on accumulation in a storage. All meteorological and hydrological observations are freely available through the web portal seklima.met.no and sildre.nve.no. To produce the gridded maps (1 km horizontal resolution), accumulated daily precipitation and daily mean temperature observations from all available measurement stations were used [9]. Gridded runoff was estimated using a hydrological rainfall-runoff model with the gridded temperature and precipitation as forcing data [10,11]. The gridded datasets of runoff, daily precipitation and daily mean temperature data are available at Senorge [12].

### 2.2 Data analysis

We conducted three analyses on the relationship between precipitation, runoff, water quality and gastroenteritis.

#### 2.2.1 Weather events (exposure) and raw/treated water quality (outcome)

Each of the meteorological and hydrological variables (exposures) were collapsed to the average of the four weeks preceding the date of the outcome variable. Water quality variables (outcomes) were collapsed to the weekly average. The observed maximum temperature was taken from the nearest meteorological station to the water source intake. For the gridded precipitation and daily mean temperature, we used the grid cell covering the intake point of the waterworks, while the gridded runoff data were calculated for the catchment area of the waterworks. The water quality data variables were subsequently merged with the meteorological and hydrological variables, by time-point and geographical location. All outcomes were transformed using log (value+1) to reduce skewness [13]. Online continuous water monitoring data had to be excluded due to unreliable sensors. For each exposure and outcome combination, we ran mixed effects linear regression models with random intercepts [14] for water work observation point and fixed effects for: month (dummy variables), internal vs external data source, and water source (surface water vs ground water vs river). We performed these procedures on all data, and then stratified by season (winter: Dec–Feb, spring: Mar–May, summer: Jun–Aug, autumn: Sep–Nov). Interaction models were used to identify if the exposure coefficients differed significantly between seasons.

We analysed effects on treated water (outcome variable) for colour and turbidity (exposure). Since the parameters, coliform bacteria, *E. coli* and intestinal enterococci mostly were reported as zero in the water quality routine monitoring data set, we were unable to run valid regression models. Bonferroni correction was applied to account for multiple testing [15].

#### 2.2.2 Raw/treated water quality (exposure) and gastroenteritis consultations (outcome)

Water quality variables (exposures) were collapsed to the average of the preceding four weeks for each municipality. The municipality averaged water quality variables were merged with the weekly outbreak (outcome) data variables, by time-point and geographical location. For each exposure, outcome, and age combination, we ran mixed effects linear regression models obtaining percentage point increase likelihood of an outbreak per unit exposure increase with random intercepts for waterworks observation point and fixed effects for month (dummy variables). We performed these procedures in all data, and then stratified by season (winter: Dec–Feb, spring: Mar–May, summer: Jun–Aug, autumn: Sep–Nov) and age (all ages, 0–4, 5–14, 15–64, 65+). We used Interaction models [16] to identify if the exposure coefficients differed significantly between seasons and/or ages. Bonferroni correction was applied to account for multiple testing [15].

#### 2.2.3 Weather (exposure) and gastroenteritis consultations (outcome)

For each combination of these exposure variables: runoff from municipal average using gridded data, rain and temperature from weather station located nearest to municipal centre; we transformed their daily values into extreme vs not-extreme using the 95th percentile (municipal specific). Then we summed these variables over a rolling four weeks period, generating variables representing the number of extreme days in a 28 days period. We subsequently analysed the exposure variables as continuous (number of extreme days in 28 days period). The municipality exposure variables were subsequently merged with the outbreak (outcome) data variables, by time-point and geographical location.

For each exposure, outcome, and age combination, we ran mixed effects linear regression models obtaining percentage point increase likelihood of an outbreak per unit exposure increase with random intercepts [14] for waterworks observation point and fixed effects for month (dummy variables). We performed these procedures in all data, and then stratified by season (winter: Dec–Feb, spring: Mar–May, summer: Jun–Aug, autumn: Sep–Nov) and age category (0–4, 5–14, 15–64, 65+). Interaction models [16] were used to identify if the exposure coefficients differed significantly between seasons and ages. Bonferroni correction [15] was applied to account for multiple testing.

## 3 Results

### 3.1 Weather and raw/treated water quality

In raw water, the interaction of the exposure with season was statistically significant for all exposures and all outcomes except for the combination of colour and temperature (Fig. C). In treated water, the interaction of the exposure with season was statistically significant for all exposures and all outcomes (Fig. C).

In raw water, increased rain and runoff were associated with increased levels of coliform bacteria, colour, *E. coli*, intestinal enterococci, and turbidity throughout the entire year with *p*-value <0.001 (Table A.1). When stratifying by season, results for rain and runoff varied, but generally acted in unison (Fig. C). In raw water, increased maximum temperature was not significantly associated with increased or decreased levels of any of the outcomes throughout the entire year, however, was associated with an increase in coliform bacteria, *E. coli*, intestinal enterococci, and turbidity in winter, and turbidity in spring (Fig. C). The main water sources in Norway are lakes, and in the last years, the period with ice-cover has decreased, probably due to changing climate. The winters are milder and are more often associated with heavy rainfall and air temperatures above zero ^o^C. The ice cover is normally a natural protection against microbes and particles flushing into the water source and prevents from an increase in microbial content and turbidity. In treated water, increased rain and runoff were associated with increased colour throughout the entire year. Increased runoff was associated with increased colour in summer and decreased colour in winter. No other associations were found (Fig. C, Table A.2).

### 3.2 Raw/treated water quality and gastroenteritis consultations (“*outbreaks*”)

An association was found between colour in treated water and increased risk of outbreaks among 0–4-year-old children with *p*-value <0.001 (Table B.1) in spring. No other significant associations between raw (Table B.2) or treated water (Table B.1) quality and outbreaks were found.

### 3.3 Weather and gastroenteritis consultations

Correlations between all ages and exposure were statistically significant (Fig. D, Table C). Interactions between seasons and exposure were statistically significant for all exposures and outcomes except for temperature that was a priori not tested, because extreme temperature events occurred primarily in summer and never in winter, which caused issues with model-fitting (Fig. D). We found that an increase in maximum temperature was associated with an increase in risk of outbreaks among all ages and 15–64 years olds for the whole year (Fig. D). We also found that an increase in rain and runoff were associated with a decrease in risk of outbreaks for different age groups and time periods throughout the year (Fig. D).

## 4 Discussion

Our results reveal a positive correlation between heavy rainfall and high runoff, and raw water quality parameters. There is large evidence in the literature about how raw water parameters get influenced by extreme weather events [4,17] which correlates well with our results. However, we did not find positive associations between increased rainfall and runoff and increase in gastroenteritis consultations (outbreaks). This is in line with our findings demonstrating no clear association between increased rainfall, runoff and treated drinking water. This suggests that the larger water works have treatment capacity that can manage changes in raw water quality due to heavy rainfall and runoff in today's climate. Interestingly, increased rain and runoff were associated with fewer consultations for gastroenteritis, while higher temperatures were associated with a higher number of more consultations. This may be linked to known risk-factors associated with good weather, such as barbecuing [18,19] and swimming in recreational water [20,21]. Regarding the small water bodies in Norway, they have been less subject to inspection and requirements for robust drinking water treatment compared to the large water works comprising of water bodies vulnerable to contamination from activities such as agriculture and surface run-off. In general, there is a requirement to protect the water sources from contamination from farms, however incidents with overflow especially with extreme weather events may be a risk. Waterborne outbreaks occur yearly, mainly caused by *Campylobacter* and norovirus where the source have been suspected to be birds or human faecal contamination. More rare cases have been linked to EHEC or *Cryptosporidium.* In case of outbreaks, a close collaboration between public health and food safety authorities is initiated for outbreak management. Recent updates in the Norwegian Drinking Water Regulation have strengthen the reporting of small-scale drinking water supply systems to the Norwegian Food Safety Authority for closer surveillance [22].

The relationship between extreme weather events and the subsequent risk of gastroenteritis due to contaminated drinking water is a complex issue [23]. In 2015, we published a review where we included analytical research studies analysing associations between extreme precipitation or temperature and waterborne disease [24]. Studies´ findings were heterogeneous. While most of them identified a positive association between increased precipitation, temperature and infection, others did not. Geographical region, season or water supply profile could play a role in this heterogeneity. Taking these findings into account, we have stratified our analysis by municipality, age, and season. However, we have not found any substantial differences suggesting, again, that effective water treatment procedures could be the reason for this.

Methodological issues, such as the definition of the outcome “waterborne disease”, could also influence research results looking into extreme weather-waterborne disease relationship. For instance, among the articles included in the above mentioned review, different types of study units were used to define “waterborne disease”: waterborne outbreaks [25,26], specific waterborne infections trends such as campylobacter infections or cholera [27], or number of gastroenteritis consultations in a health care facility [28]. All publications studying waterborne outbreaks (*n* = 4) found an association between precipitation and waterborne disease, while findings in those using single cases of infection or consultations were more heterogeneous (*n* = 20),. In 2016, a previous collaboration between NIPH, MET and NVE together with national public health institutes from three additional Nordic countries and the European Centre for Disease Prevention and Control examined the association between heavy precipitation events and waterborne outbreaks between 1992 and 2012 using waterborne outbreaks as study units [29]. We did find a positive association between increased precipitation during the preceding week and the occurrence of an outbreak, specifically involving single household supplies. Outbreaks associated with waterworks did not present a significant correlation with heavy precipitation events, which is in line with the results of our current study. The conclusions from the previous project were somehow limited as underreporting is an inherent problem in surveillance systems and notified outbreaks are the tip of the iceberg of the real burden of waterborne disease. Even with the use of more sensitive registry based on syndromic surveillance, NorSySS, we found no evidence for an association between extreme weather events and gastroenteritis consultations. A potential limitation of this study, could be that gastroenteritis consultations included in NorSySS are an aggregated large group of consultation from which we cannot disentangle those that are waterborne, which may dilute the potential relationship we are assessing.

For the first analysis in this study, each of the meteorological, hydrological, and water quality variables were collapsed to averages for each location, before the associations between weather, hydrology and water quality indicators were estimated. As contaminations of the drinking water are expected to occur after heavy rainfall events, using “number of days in a week where the meteorological and hydrological variable was above the 95th percentile” was considered. However, weekly averages provided very similar results. The associations found between water quality and weekly average rainfall therefore also indicate associations between water quality and a high frequency of days with extreme precipitation.

The spatial pattern of rainfall is highly variable due to complex relationships between climate regimes, seasonality, and topography. To capture this variability, it demands a high-resolution network of measurement stations. Levy et al. [30] found that spatial incompatibility between exposed populations and rain gauges in their study resulted in a reduction of about 50% in the association between extreme rainfall and diarrheal disease incidence. Although the observational network in Norway used in the gridded datasets consists of several hundred weather stations and gives a good representation of the regional climate, there is uncertainty in the estimates for the exposure location. As the gridded climate data is used as input to a conceptual rainfall-runoff model to estimate runoff, this uncertainty is also embedded in the runoff data. In our study, we used daily accumulated precipitation and daily mean temperature as a basis for the calculations. Higher time resolutions would have better captured extreme rainfall events that occur over a short duration, but only a limited number of such datasets exist. However, as we aggregate the data to a weekly time resolution, this is not believed to have a major impact on the results. The lack of exact weather representations at the exposure locations, in combination with limited access to data about the exact locations of the intake points for the waterworks, due to security reasons, may have led to a decrease in potential associations between heavy rainfall events and drinking water quality.

## 5 Conclusions

Our results illustrate that larger water works in Norway seem to cope with those extreme weather events that we have experienced so far. However, we have not been able to assess small waterworks and single households in this study as they do not collect enough data on raw or treated water quality to be able to do an analysis of the association.

Aging drinking water treatment, distribution systems and sewage systems will be particularly vulnerable to flooding, leading to potential deterioration in the quality of drinking water. Therefore, we also perform work to assess how future climate scenarios will challenge raw water quality and the water treatment capacity in a follow up study that is a part of the same research project. Using the data from this study and future climate scenarios combined with modelling of treatment effect and QMRA [31] to assess probability of disease is needed to address and prepare for a changing climate with more extreme weather events and potential need for increased treatment capacity. This will be central to minimize the risk and burden of waterborne disease in the future.

Based on our results, water safety planners, in areas using surface water as a source for drinking water production, should consider including coagulation and UV radiation as treatment, as it appears as robust for changes in water quality due to extreme weather events. However, treated water also may be contaminated during distribution to the consumers. Therefore, effects of extreme weather events associated with drinking water distribution systems and waterborne disease needs to be further explored. The One Health link in this study could be a marker for future studies, in the facilitation of human health, veterinary and environmental aspects to shed light of an emerging health issue concerning climate change, water and health.

## Funding

This work was supported by the 10.13039/501100005416Norwegian Research Council [No. 244147/E10].

## Declaration of Competing Interest

The authors declare that they have no known competing financial interests or personal relationships that could have appeared to influence the work reported in this paper.

## Figures and Tables

**Fig. A:**
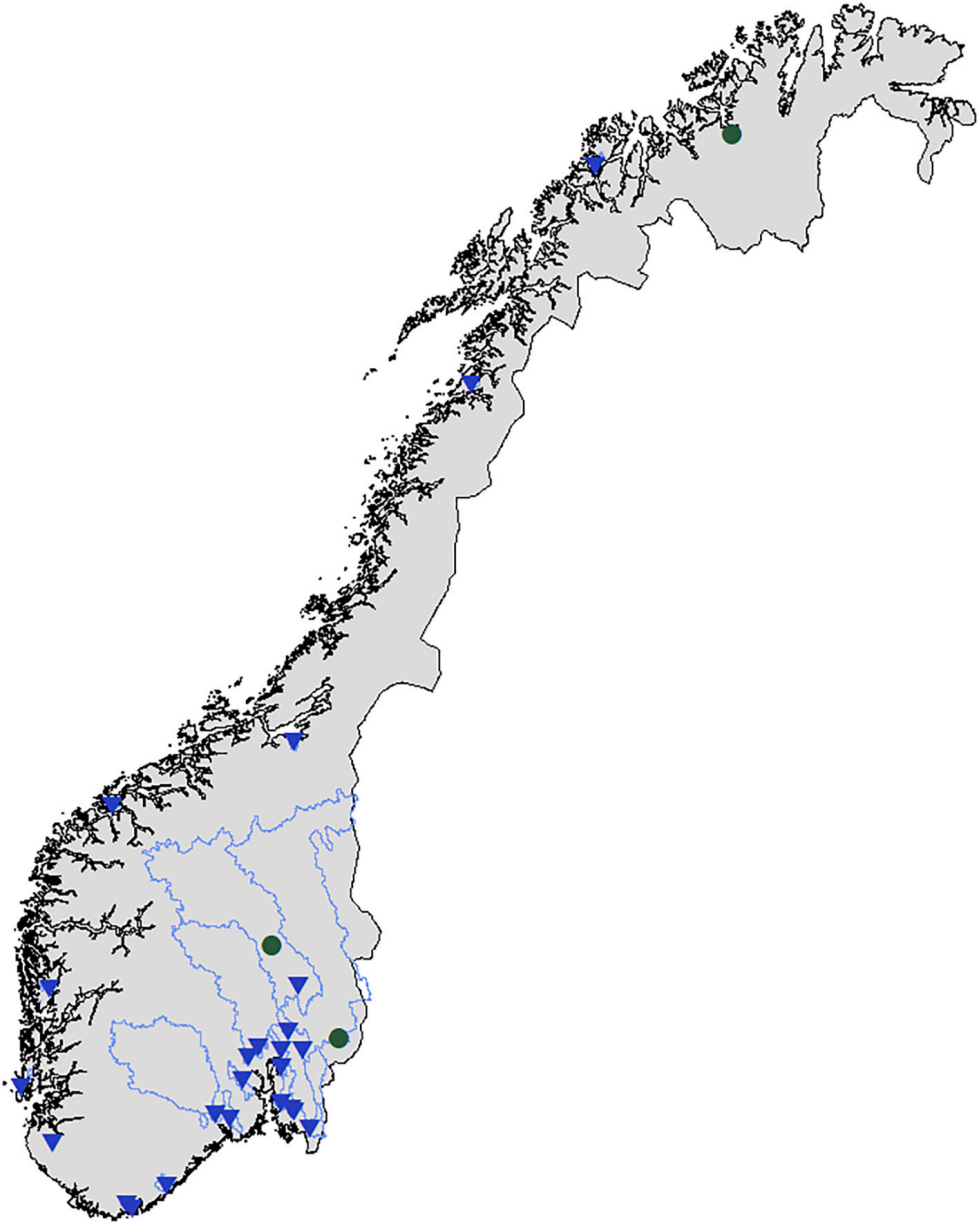
Location of the waterworks used in this study. Groundwater works are indicated by the dots, whereas surface water works are represented by the inverted triangles. The light blue colour shows the catchment boundaries. (For interpretation of the references to colour in this figure legend, the reader is referred to the web version of this article.)

**Fig. B:**
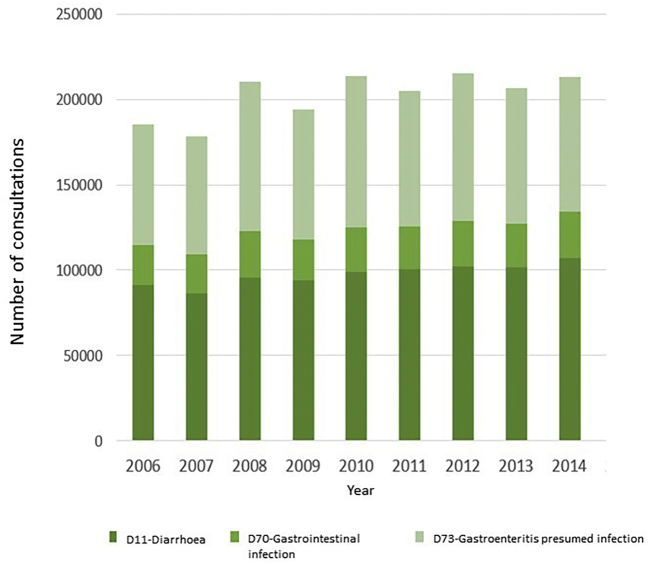
Number of consultations for gastrointestinal infections 2006–2014.

**Fig. C:**
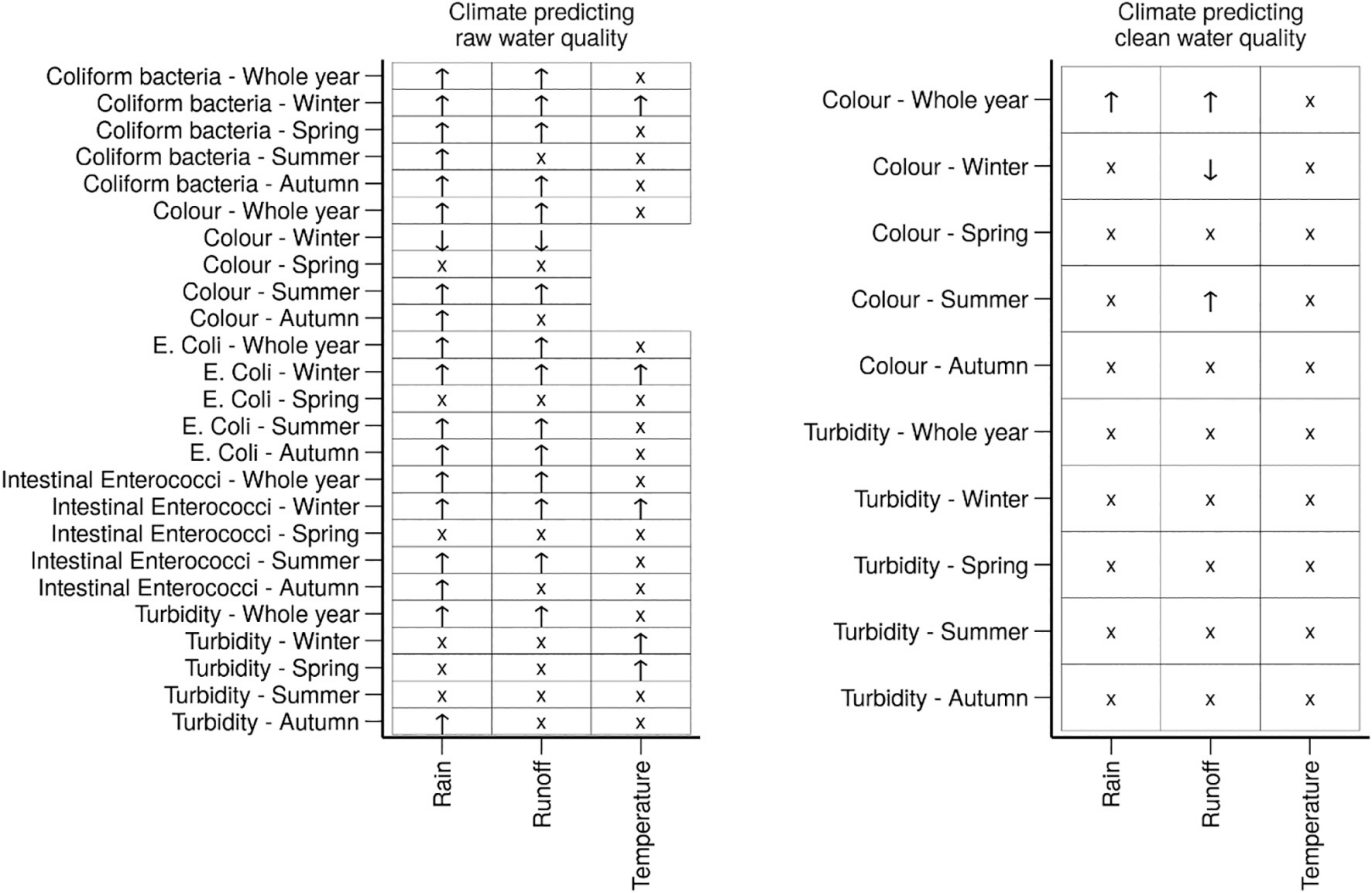
Association between extreme weather events, and raw or clean (treated/distributed from waterworks) water quality in selected waterworks.

**Fig. D:**
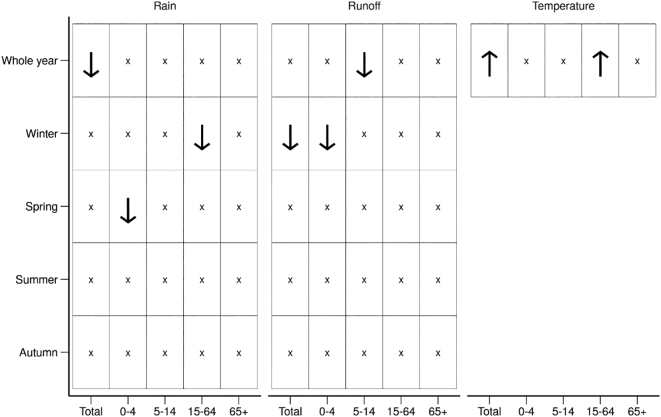
Association between extreme weather events (exposure), and gastroenteritis outbreaks (outcome).

**Table A.1:** Raw water.

Season	Outcome	Exposure	Marginal_r2	n	Effect	Coef	Coef_se	Pval	Pvalbonf	interactionpvalseason
Whole year	Coliform bacteria	c_gridRain0_3	0.187849505	9061	6.47% (5.61%, 7.34%)	0.062712813	0.004145672	<0.001	<0.001*	<0.001
Whole year	Coliform bacteria	c_gridRunoffStandardised0_3	0.175918459	9061	4.20% (3.50%, 4.91%)	0.041177825	0.003460164	<0.001	<0.001*	<0.001
Whole year	Coliform bacteria	c_temperature0_3	0.164593553	9061	0.88% (−0.07%, 1.84%)	0.008793641	0.00483196	0.069	1.000	<0.001
Whole year	Colour	c_gridRain0_3	0.038798323	8215	0.91% (0.72%, 1.10%)	0.009047818	0.000960882	<0.001	<0.001*	<0.001
Whole year	Colour	c_gridRunoffStandardised0_3	0.038148825	8215	0.85% (0.68%, 1.02%)	0.008466735	0.000841661	<0.001	<0.001*	<0.001
Whole year	Colour	c_temperature0_3	0.03902032	8215	−0.12% (−0.33%, 0.10%)	−0.001188186	0.001091158	0.276	1.000	0.085
Whole year	*E. Coli*	c_gridRain0_3	0.246384334	9036	4.21% (3.68%, 4.74%)	0.04125476	0.002592617	<0.001	<0.001*	<0.001
Whole year	*E. Coli*	c_gridRunoffStandardised0_3	0.234934884	9036	2.47% (2.04%, 2.89%)	0.024353863	0.002121482	<0.001	<0.001*	<0.001
Whole year	*E. Coli*	c_temperature0_3	0.233388639	9036	−0.24% (−0.82%, 0.35%)	−0.002406732	0.002990361	0.421	1.000	0.027
Whole year	Intestinal Enterococci	c_gridRain0_3	0.294010564	6316	1.52% (1.17%, 1.87%)	0.015074312	0.001747859	<0.001	<0.001*	<0.001
Whole year	Intestinal Enterococci	c_gridRunoffStandardised0_3	0.28917412	6316	0.77% (0.48%, 1.06%)	0.007691455	0.001480288	<0.001	<0.001*	0.025
Whole year	Intestinal Enterococci	c_temperature0_3	0.289093123	6316	0.15% (−0.26%, 0.56%)	0.001454267	0.002088931	0.486	1.000	0.001
Whole year	Turbidity	c_gridRain0_3	0.093994433	8502	0.60% (0.40%, 0.81%)	0.006012897	0.001032193	<0.001	<0.001*	<0.001
Whole year	Turbidity	c_gridRunoffStandardised0_3	0.093321221	8502	0.41% (0.24%, 0.58%)	0.004126737	0.00086094	<0.001	<0.001*	<0.001
Whole year	Turbidity	c_temperature0_3	0.092778213	8502	0.26% (0.03%, 0.49%)	0.002620951	0.001161909	0.024	1.000	<0.001
Winter	Coliform bacteria	c_gridRain0_3	0.170513048	2093	4.51% (3.29%, 5.74%)	0.044099817	0.005998201	<0.001	<0.001*	NA
Winter	Coliform bacteria	c_gridRunoffStandardised0_3	0.164793156	2093	3.37% (2.23%, 4.53%)	0.033164798	0.005672744	<0.001	<0.001*	NA
Winter	Coliform bacteria	c_temperature0_3	0.172493856	2093	6.65% (5.12%, 8.20%)	0.064399208	0.007374063	<0.001	<0.001*	NA
Winter	Colour	c_gridRain0_3	0.043609035	1933	−0.73% (−1.05%, −0.41%)	−0.007346422	0.001656446	<0.001	0.001*	NA
Winter	Colour	c_gridRunoffStandardised0_3	0.045895443	1933	−0.90% (−1.20%, −0.60%)	−0.009061083	0.001554928	<0.001	<0.001*	NA
Winter	Colour	c_temperature0_3	0.039489338	1933	−0.16% (−0.54%, 0.22%)	−0.001616098	0.001960851	0.410	1.000	NA
Winter	*E. Coli*	c_gridRain0_3	0.215764324	2082	3.26% (2.46%, 4.06%)	0.032060928	0.003966521	<0.001	<0.001*	NA
Winter	*E. Coli*	c_gridRunoffStandardised0_3	0.215117155	2082	2.92% (2.17%, 3.67%)	0.028750231	0.003714684	<0.001	<0.001*	NA
Winter	*E. Coli*	c_temperature0_3	0.215012196	2082	3.20% (2.22%, 4.20%)	0.031522187	0.004902143	<0.001	<0.001*	NA
Winter	Intestinal Enterococci	c_gridRain0_3	0.309159381	1489	2.01% (1.40%, 2.62%)	0.019890746	0.003056931	<0.001	<0.001*	NA
Winter	Intestinal Enterococci	c_gridRunoffStandardised0_3	0.303030882	1489	1.25% (0.68%, 1.82%)	0.012399872	0.002860907	<0.001	0.001*	NA
Winter	Intestinal Enterococci	c_temperature0_3	0.3096354	1489	2.58% (1.79%, 3.38%)	0.025493727	0.003933506	<0.001	<0.001*	NA
Winter	Turbidity	c_gridRain0_3	0.042250325	1975	0.53% (0.19%, 0.88%)	0.005331226	0.001757078	0.002	0.181	NA
Winter	Turbidity	c_gridRunoffStandardised0_3	0.041261829	1975	0.30% (−0.02%, 0.63%)	0.003023848	0.001665041	0.069	1.000	NA
Winter	Turbidity	c_temperature0_3	0.0483697	1975	1.58% (1.18%, 1.99%)	0.015706971	0.002030734	<0.001	<0.001*	NA
Spring	Coliform bacteria	c_gridRain0_3	0.180720353	2243	7.85% (6.30%, 9.42%)	0.07555685	0.007393668	<0.001	<0.001*	NA
Spring	Coliform bacteria	c_gridRunoffStandardised0_3	0.169042184	2243	4.49% (3.37%, 5.62%)	0.043906177	0.005477325	<0.001	<0.001*	NA
Spring	Coliform bacteria	c_temperature0_3	0.156956216	2243	1.81% (0.41%, 3.24%)	0.017972393	0.007092333	0.011	0.846	NA
Spring	Colour	c_gridRain0_3	0.036119979	1958	−0.30% (−0.73%, 0.13%)	−0.002997044	0.002185204	0.170	1.000	NA
Spring	Colour	c_gridRunoffStandardised0_3	0.035672174	1958	−0.11% (−0.44%, 0.21%)	−0.001130361	0.001669815	0.498	1.000	NA
Spring	Colour	c_temperature0_3	0.036003542	1958	0.38% (−0.01%, 0.76%)	0.003745235	0.001956525	0.056	1.000	NA
Spring	*E. Coli*	c_gridRain0_3	0.261908323	2232	1.00% (0.03%, 1.99%)	0.009964393	0.00495099	0.044	1.000	NA
Spring	*E. Coli*	c_gridRunoffStandardised0_3	0.263098054	2232	0.52% (−0.18%, 1.22%)	0.005160457	0.003576674	0.149	1.000	NA
Spring	*E. Coli*	c_temperature0_3	0.267765379	2232	0.96% (0.06%, 1.86%)	0.009509531	0.004545525	0.036	1.000	NA
Spring	Intestinal Enterococci	c_gridRain0_3	0.438274821	1559	0.38% (−0.25%, 1.01%)	0.003771575	0.003193121	0.238	1.000	NA
Spring	Intestinal Enterococci	c_gridRunoffStandardised0_3	0.437823899	1559	0.28% (−0.18%, 0.75%)	0.002827486	0.002367935	0.232	1.000	NA
Spring	Intestinal Enterococci	c_temperature0_3	0.440076256	1559	0.14% (−0.48%, 0.76%)	0.001400196	0.003165922	0.658	1.000	NA
Spring	Turbidity	c_gridRain0_3	0.110625568	2048	0.55% (0.06%, 1.05%)	0.005519537	0.002500823	0.027	1.000	NA
Spring	Turbidity	c_gridRunoffStandardised0_3	0.110401163	2048	0.53% (0.16%, 0.90%)	0.005274118	0.001869052	0.005	0.358	NA
Spring	Turbidity	c_temperature0_3	0.114512166	2048	1.19% (0.75%, 1.63%)	0.011798486	0.002220313	<0.001	<0.001*	NA
Summer	Coliform bacteria	c_gridRain0_3	0.132159991	2365	5.11% (2.54%, 7.73%)	0.049790922	0.012595315	<0.001	0.006*	NA
Summer	Coliform bacteria	c_gridRunoffStandardised0_3	0.126266182	2365	3.54% (1.38%, 5.75%)	0.034811287	0.010747361	0.001	0.090	NA
Summer	Coliform bacteria	c_temperature0_3	0.125492343	2365	−1.14% (−3.94%, 1.74%)	−0.011469155	0.01466393	0.434	1.000	NA
Summer	Colour	c_gridRain0_3	0.02183259	2145	1.05% (0.59%, 1.51%)	0.010436338	0.002327594	<0.001	0.001*	NA
Summer	Colour	c_gridRunoffStandardised0_3	0.020588564	2145	1.22% (0.78%, 1.66%)	0.012136531	0.002212337	<0.001	<0.001*	NA
Summer	Colour	c_temperature0_3	0.021126713	2145	−1.24% (−1.77%, −0.71%)	−0.012471016	0.002731452	<0.001	<0.001*	NA
Summer	*E. Coli*	c_gridRain0_3	0.249025578	2359	8.36% (6.85%, 9.90%)	0.08032603	0.007201048	<0.001	<0.001*	NA
Summer	*E. Coli*	c_gridRunoffStandardised0_3	0.218343151	2359	4.66% (3.42%, 5.91%)	0.045511541	0.006081242	<0.001	<0.001*	NA
Summer	*E. Coli*	c_temperature0_3	0.214963055	2359	−1.65% (−3.26%, −0.01%)	−0.016665343	0.008429973	0.048	1.000	NA
Summer	Intestinal Enterococci	c_gridRain0_3	0.232905675	1634	2.74% (1.85%, 3.62%)	0.026988072	0.004396277	<0.001	<0.001*	NA
Summer	Intestinal Enterococci	c_gridRunoffStandardised0_3	0.220084512	1634	1.29% (0.55%, 2.04%)	0.012824195	0.003751988	0.001	0.047*	NA
Summer	Intestinal Enterococci	c_temperature0_3	0.215686619	1634	−0.42% (−1.41%, 0.58%)	−0.004194729	0.005112795	0.412	1.000	NA
Summer	Turbidity	c_gridRain0_3	0.09709874	2229	0.40% (−0.07%, 0.88%)	0.004040483	0.00241477	0.094	1.000	NA
Summer	Turbidity	c_gridRunoffStandardised0_3	0.096884836	2229	−0.00% (−0.40%, 0.40%)	−2.47704E-05	0.002048832	0.990	1.000	NA
Summer	Turbidity	c_temperature0_3	0.097031911	2229	0.19% (−0.36%, 0.74%)	0.001863681	0.002794215	0.505	1.000	NA
Autumn	Coliform bacteria	c_gridRain0_3	0.08265564	2360	5.23% (3.59%, 6.90%)	0.050998234	0.008027474	<0.001	<0.001*	NA
Autumn	Coliform bacteria	c_gridRunoffStandardised0_3	0.080889408	2360	4.46% (2.77%, 6.18%)	0.043654809	0.008319847	<0.001	<0.001*	NA
Autumn	Coliform bacteria	c_temperature0_3	0.065680555	2360	0.08% (−2.22%, 2.44%)	0.000844424	0.011859919	0.943	1.000	NA
Autumn	Colour	c_gridRain0_3	0.038077979	2179	0.73% (0.40%, 1.07%)	0.007320732	0.001673919	<0.001	0.001*	NA
Autumn	Colour	c_gridRunoffStandardised0_3	0.037473832	2179	0.55% (0.21%, 0.90%)	0.005533131	0.00174828	0.002	0.116	NA
Autumn	Colour	c_temperature0_3	0.037803865	2179	−0.70% (−1.17%, −0.22%)	−0.006982164	0.002439641	0.004	0.316	NA
Autumn	*E. Coli*	c_gridRain0_3	0.195780485	2363	4.77% (3.76%, 5.78%)	0.046564311	0.004911303	<0.001	<0.001*	NA
Autumn	*E. Coli*	c_gridRunoffStandardised0_3	0.182324764	2363	3.48% (2.46%, 4.51%)	0.034194748	0.005071137	<0.001	<0.001*	NA
Autumn	*E. Coli*	c_temperature0_3	0.166833811	2363	−0.50% (−1.89%, 0.91%)	−0.004975076	0.007171812	0.488	1.000	NA
Autumn	Intestinal Enterococci	c_gridRain0_3	0.228923373	1634	1.79% (1.06%, 2.52%)	0.017736331	0.003666347	<0.001	<0.001*	NA
Autumn	Intestinal Enterococci	c_gridRunoffStandardised0_3	0.218163507	1634	0.73% (0.01%, 1.45%)	0.007262297	0.003642395	0.046	1.000	NA
Autumn	Intestinal Enterococci	c_temperature0_3	0.216438983	1634	−0.62% (−1.70%, 0.47%)	−0.006236368	0.005587882	0.264	1.000	NA
Autumn	Turbidity	c_gridRain0_3	0.136836478	2250	0.80% (0.46%, 1.14%)	0.007967024	0.001740062	<0.001	<0.001*	NA
Autumn	Turbidity	c_gridRunoffStandardised0_3	0.132318997	2250	0.30% (−0.05%, 0.66%)	0.003017987	0.001817705	0.097	1.000	NA
Autumn	Turbidity	c_temperature0_3	0.131392966	2250	−0.40% (−0.89%, 0.09%)	−0.004010076	0.002514244	0.111	1.000	NA

**Table A.2:** Treated water.

Season	Outcome	Exposure	Marginal_r2	n	Effect	Coef	Coef_se	Pval	Pvalbonf	Interaction Pval Season
Whole year	Colour	c_gridRain0_3	0.173208356	10,832	0.40% (0.17%, 0.64%)	0.0040242	0.0011816	0.001	0.020*	<0.001
Whole year	Colour	c_gridRunoffStandardised0_3	0.173476069	10,832	0.34% (0.15%, 0.54%)	0.003414	0.00099	0.001	0.017*	<0.001
Whole year	Colour	c_temperature0_3	0.08367322	9752	−0.22% (−0.47%, 0.04%)	−0.002165	0.0013225	0.102	1.000	<0.001
Whole year	Turbidity	c_gridRain0_3	0.03804621	11,243	−0.02% (−0.09%, 0.05%)	−0.000228	0.0003533	0.519	1.000	<0.001
Whole year	Turbidity	c_gridRunoffStandardised0_3	0.039457797	11,243	0.03% (−0.03%, 0.09%)	0.0003132	0.0002959	0.290	1.000	<0.001
Whole year	Turbidity	c_temperature0_3	0.046433938	10,096	−0.05% (−0.12%, 0.03%)	−0.000472	0.000387	0.223	1.000	0.015
Winter	Colour	c_gridRain0_3	0.164647808	2522	−0.52% (−0.94%, −0.10%)	−0.005207	0.002153	0.016	0.468	NA
Winter	Colour	c_gridRunoffStandardised0_3	0.166448158	2522	−0.72% (−1.12%, −0.32%)	−0.007213	0.0020427	<0.001	0.012*	NA
Winter	Colour	c_temperature0_3	0.075798426	2253	−0.48% (−0.98%, 0.02%)	−0.004837	0.0025454	0.057	1.000	NA
Winter	Turbidity	c_gridRain0_3	0.024764078	2619	−0.08% (−0.23%, 0.06%)	−0.00083	0.0007457	0.266	1.000	NA
Winter	Turbidity	c_gridRunoffStandardised0_3	0.024916535	2619	−0.11% (−0.25%, 0.03%)	−0.001087	0.000705	0.123	1.000	NA
Winter	Turbidity	c_temperature0_3	0.04151168	2337	−0.06% (−0.22%, 0.09%)	−0.000628	0.0008033	0.434	1.000	NA
Spring	Colour	c_gridRain0_3	0.153908343	2681	−0.60% (−1.19%, −0.02%)	−0.006053	0.0030085	0.044	1.000	NA
Spring	Colour	c_gridRunoffStandardised0_3	0.153118641	2681	−0.38% (−0.80%, 0.04%)	−0.003804	0.0021551	0.078	1.000	NA
Spring	Colour	c_temperature0_3	0.076473509	2413	−0.36% (−0.88%, 0.16%)	−0.003614	0.0026715	0.176	1.000	NA
Spring	Turbidity	c_gridRain0_3	0.03434081	2769	−0.08% (−0.28%, 0.12%)	−0.000804	0.0010435	0.441	1.000	NA
Spring	Turbidity	c_gridRunoffStandardised0_3	0.035893508	2769	0.04% (−0.10%, 0.19%)	0.000447	0.0007439	0.548	1.000	NA
Spring	Turbidity	c_temperature0_3	0.040554238	2494	0.10% (−0.09%, 0.28%)	0.0009527	0.0009419	0.312	1.000	NA
Summer	Colour	c_gridRain0_3	0.186397469	2822	0.27% (−0.38%, 0.93%)	0.0027362	0.0033403	0.413	1.000	NA
Summer	Colour	c_gridRunoffStandardised0_3	0.189230852	2822	1.17% (0.59%, 1.75%)	0.0116039	0.0029176	<0.001	0.002*	NA
Summer	Colour	c_temperature0_3	0.09003993	2549	−0.02% (−0.77%, 0.74%)	−0.000181	0.0038609	0.963	1.000	NA
Summer	Turbidity	c_gridRain0_3	0.063709584	2924	−0.03% (−0.18%, 0.11%)	−0.000332	0.0007305	0.649	1.000	NA
Summer	Turbidity	c_gridRunoffStandardised0_3	0.065369953	2924	0.02% (−0.10%, 0.15%)	0.0002288	0.0006306	0.717	1.000	NA
Summer	Turbidity	c_temperature0_3	0.066981165	2636	−0.02% (−0.18%, 0.15%)	−0.000197	0.0008441	0.816	1.000	NA
Autumn	Colour	c_gridRain0_3	0.174733242	2807	0.29% (−0.12%, 0.71%)	0.0029105	0.0021159	0.169	1.000	NA
Autumn	Colour	c_gridRunoffStandardised0_3	0.174201321	2807	−0.25% (−0.68%, 0.18%)	−0.00249	0.0022092	0.260	1.000	NA
Autumn	Colour	c_temperature0_3	0.071057911	2537	−0.43% (−0.99%, 0.15%)	−0.004267	0.0029239	0.144	1.000	NA
Autumn	Turbidity	c_gridRain0_3	0.040858814	2931	−0.00% (−0.11%, 0.11%)	−5.71E-06	0.0005537	0.992	1.000	NA
Autumn	Turbidity	c_gridRunoffStandardised0_3	0.039348177	2931	−0.05% (−0.16%, 0.06%)	−0.000486	0.0005713	0.395	1.000	NA
Autumn	Turbidity	c_temperature0_3	0.041906822	2629	−0.05% (−0.21%, 0.10%)	−0.00054	0.0007735	0.485	1.000	NA

**Table B.1:** Treated water and outbreaks.

Age	Season	Outcome	Outcome summary	Exposure	Exposure Summary	Marginal_r2	n	Effect	Coef	Coef_se	Pval	PvalBonf	Interaction PvalAge	Interaction PvalSeason
Totalt	Whole year	Outbreak	6.03%	Colour	50p = 5.50, 75p = 7.97, 95p = 14.25	0.0243468	10,738	0.03 pp. (−0.10 pp., 0.17 pp)	0.000328277	0.0006747	0.627	1.000	<0.001	<0.001
Totalt	Whole year	Outbreak	6.02%	Turbidity	50p = 0.13, 75p = 0.18, 95p = 0.34	0.0244193	10,738	−1.59 pp. (−3.95 pp., 0.77 pp)	−0.01589816	0.0120322	0.186	1.000	<0.001	0.001
Totalt	Winter	Outbreak	7.22%	Colour	50p = 5.75, 75p = 8.60, 95p = 14.25	0.0029938	2341	−0.28 pp. (−0.60 pp., 0.04 pp)	−0.00282424	0.0016386	0.085	1.000	NA	NA
Totalt	Winter	Outbreak	7.19%	Turbidity	50p = 0.13, 75p = 0.20, 95p = 0.39	0.0031661	2349	−5.55 pp. (−10.54 pp., −0.55 pp)	−0.05545235	0.0254674	0.029	1.000	NA	NA
Totalt	Spring	Outbreak	5.49%	Colour	50p = 5.50, 75p = 7.83, 95p = 14.50	0.0215318	2732	0.28 pp. (0.02 pp., 0.54 pp)	0.002824032	0.001334	0.034	1.000	NA	NA
Totalt	Spring	Outbreak	5.38%	Turbidity	50p = 0.13, 75p = 0.19, 95p = 0.34	0.0196242	2732	2.41 pp. (−1.79 pp., 6.60 pp)	0.024052745	0.0213921	0.261	1.000	NA	NA
Totalt	Summer	Outbreak	8.54%	Colour	50p = 5.00, 75p = 7.72, 95p = 14.00	0.0323312	2809	0.03 pp. (−0.34 pp., 0.39 pp)	0.000272482	0.001876	0.885	1.000	NA	NA
Totalt	Summer	Outbreak	8.57%	Turbidity	50p = 0.13, 75p = 0.17, 95p = 0.31	0.0329439	2800	−1.78 pp. (−6.24 pp., 2.68 pp)	−0.01776393	0.0227553	0.435	1.000	NA	NA
Totalt	Autumn	Outbreak	3.12%	Colour	50p = 6.12, 75p = 8.50, 95p = 14.00	0.0013191	2856	−0.07 pp. (−0.26 pp., 0.11 pp)	−0.00073282	0.0009531	0.442	1.000	NA	NA
Totalt	Autumn	Outbreak	3.15%	Turbidity	50p = 0.12, 75p = 0.17, 95p = 0.31	0.0009583	2857	1.10 pp. (−6.52 pp., 8.73 pp)	0.011042823	0.0389126	0.777	1.000	NA	NA
65+	Whole year	Outbreak	5.07%	Colour	50p = 5.50, 75p = 7.97, 95p = 14.25	0.0056343	10,737	−0.12 pp. (−0.24 pp., −0.01 pp)	−0.00124375	0.0005979	0.038	1.000	NA	NA
65+	Whole year	Outbreak	5.03%	Turbidity	50p = 0.13, 75p = 0.18, 95p = 0.34	0.0055919	10,737	−0.01 pp. (−2.19 pp., 2.17 pp)	−7.7188E−05	0.0111212	0.994	1.000	NA	NA
65+	Winter	Outbreak	5.04%	Colour	50p = 5.75, 75p = 8.60, 95p = 14.25	0.0002137	2341	0.03 pp. (−0.22 pp., 0.28 pp)	0.000307196	0.0012562	0.807	1.000	NA	NA
65+	Winter	Outbreak	5.02%	Turbidity	50p = 0.13, 75p = 0.20, 95p = 0.39	0.0013035	2349	3.46 pp. (−0.75 pp., 7.66 pp)	0.034583118	0.0214456	0.107	1.000	NA	NA
65+	Spring	Outbreak	4.50%	Colour	50p = 5.50, 75p = 7.83, 95p = 14.50	0.0046968	2732	-0.16 pp. (−0.38 pp., 0.06 pp)	−0.00160939	0.0011033	0.145	1.000	NA	NA
65+	Spring	Outbreak	4.39%	Turbidity	50p = 0.13, 75p = 0.19, 95p = 0.34	0.004338	2732	−1.83 pp. (−5.67 pp., 2.00 pp)	−0.0183455	0.019565	0.348	1.000	NA	NA
65+	Summer	Outbreak	6.55%	Colour	50p = 5.00, 75p = 7.72, 95p = 14.00	0.0068543	2809	−0.15 pp. (−0.47 pp., 0.17 pp)	−0.0015063	0.0016548	0.363	1.000	NA	NA
65+	Summer	Outbreak	6.57%	Turbidity	50p = 0.13, 75p = 0.17, 95p = 0.31	0.0073183	2800	−1.50 pp. (−5.50 pp., 2.49 pp)	−0.01501281	0.0203778	0.461	1.000	NA	NA
65+	Autumn	Outbreak	4.17%	Colour	50p = 6.12, 75p = 8.50, 95p = 14.00	0.0047943	2855	−0.34 pp. (−0.59 pp., −0.09 pp)	−0.00337596	0.0012725	0.008	0.399	NA	NA
65+	Autumn	Outbreak	4.13%	Turbidity	50p = 0.12, 75p = 0.17, 95p = 0.31	0.0012808	2856	0.02 pp. (−9.29 pp., 9.34 pp)	0.00023116	0.0475236	0.996	1.000	NA	NA
5–14	Whole year	Outbreak	5.26%	Colour	50p = 5.50, 75p = 7.96, 95p = 14.25	0.0077854	10,708	0.06 pp. (−0.05 pp., 0.18 pp)	0.000647847	0.0006086	0.287	1.000	NA	NA
5–14	Whole year	Outbreak	5.27%	Turbidity	50p = 0.13, 75p = 0.18, 95p = 0.34	0.0077063	10,708	−0.50 pp. (−2.73 pp., 1.73 pp)	−0.00498053	0.011365	0.661	1.000	NA	NA
5–14	Winter	Outbreak	4.24%	Colour	50p = 5.75, 75p = 8.60, 95p = 14.25	0.0026064	2336	0.27 pp. (0.06 pp., 0.49 pp)	0.002749733	0.0011168	0.014	0.691	NA	NA
5–14	Winter	Outbreak	4.27%	Turbidity	50p = 0.13, 75p = 0.20, 95p = 0.39	0.0002557	2344	−1.39 pp. (−5.27 pp., 2.49 pp)	−0.01391713	0.019811	0.482	1.000	NA	NA
5–14	Spring	Outbreak	5.54%	Colour	50p = 5.50, 75p = 7.82, 95p = 14.50	0.0067	2726	0.13 pp. (−0.15 pp., 0.41 pp)	0.001294515	0.0014162	0.361	1.000	NA	NA
5–14	Spring	Outbreak	5.50%	Turbidity	50p = 0.13, 75p = 0.19, 95p = 0.34	0.0062901	2726	0.87 pp. (−3.43 pp., 5.17 pp)	0.008690457	0.0219287	0.692	1.000	NA	NA
5–14	Summer	Outbreak	6.97%	Colour	50p = 5.00, 75p = 7.72, 95p = 14.00	0.0090417	2798	−0.09 pp. (−0.37 pp., 0.18 pp)	−0.0009177	0.0013983	0.512	1.000	NA	NA
5–14	Summer	Outbreak	6.99%	Turbidity	50p = 0.13, 75p = 0.17, 95p = 0.31	0.0090928	2789	−0.20 pp. (−4.26 pp., 3.86 pp)	−0.00198601	0.0207215	0.924	1.000	NA	NA
5–14	Autumn	Outbreak	4.14%	Colour	50p = 6.12, 75p = 8.47, 95p = 14.00	0.0012682	2848	0.04 pp. (−0.19 pp., 0.28 pp)	0.000429029	0.0011911	0.719	1.000	NA	NA
5–14	Autumn	Outbreak	4.18%	Turbidity	50p = 0.12, 75p = 0.17, 95p = 0.31	0.002043	2849	−6.39 pp. (−15.42 pp., 2.65 pp)	−0.06387795	0.0461078	0.166	1.000	NA	NA
15–64	Whole year	Outbreak	5.81%	Colour	50p = 5.50, 75p = 7.97, 95p = 14.25	0.0301294	10,738	−0.11 pp. (−0.26 pp., 0.03 pp)	−0.00114137	0.0007528	0.129	1.000	NA	NA
15–64	Whole year	Outbreak	5.83%	Turbidity	50p = 0.13, 75p = 0.18, 95p = 0.34	0.0303317	10,738	−1.91 pp. (−4.24 pp., 0.42 pp)	−0.01912747	0.0118942	0.108	1.000	NA	NA
15–64	Winter	Outbreak	7.60%	Colour	50p = 5.75, 75p = 8.60, 95p = 14.25	0.0038985	2341	−0.39 pp. (−0.71 pp., −0.06 pp)	−0.00387697	0.0016468	0.019	0.928	NA	NA
15–64	Winter	Outbreak	7.58%	Turbidity	50p = 0.13, 75p = 0.20, 95p = 0.39	0.0023544	2349	−4.73 pp. (−9.84 pp., 0.38 pp)	−0.04730557	0.0260527	0.069	1.000	NA	NA
15–64	Spring	Outbreak	3.18%	Colour	50p = 5.50, 75p = 7.83, 95p = 14.50	0.0085432	2732	0.02 pp. (−0.19 pp., 0.22 pp)	0.000185726	0.0010434	0.859	1.000	NA	NA
15–64	Spring	Outbreak	3.15%	Turbidity	50p = 0.13, 75p = 0.19, 95p = 0.34	0.0082262	2732	0.25 pp. (−3.03 pp., 3.53 pp)	0.002508301	0.0167459	0.881	1.000	NA	NA
15–64	Summer	Outbreak	9.75%	Colour	50p = 5.00, 75p = 7.72, 95p = 14.00	0.0294026	2809	−0.06 pp. (−0.49 pp., 0.37 pp)	−0.00056405	0.0021897	0.797	1.000	NA	NA
15–64	Summer	Outbreak	9.82%	Turbidity	50p = 0.13, 75p = 0.17, 95p = 0.31	0.030125	2800	−1.15 pp. (−5.90 pp., 3.61 pp)	−0.01147295	0.0242616	0.636	1.000	NA	NA
15–64	Autumn	Outbreak	2.98%	Colour	50p = 6.12, 75p = 8.50, 95p = 14.00	0.0008909	2856	−0.05 pp. (−0.24 pp., 0.13 pp)	−0.00054953	0.0009535	0.564	1.000	NA	NA
15–64	Autumn	Outbreak	3.05%	Turbidity	50p = 0.12, 75p = 0.17, 95p = 0.31	0.0007551	2857	1.36 pp. (−6.14 pp., 8.87 pp)	0.013636286	0.0382832	0.722	1.000	NA	NA
0–4	Whole year	Outbreak	5.16%	Colour	50p = 5.50, 75p = 7.96, 95p = 14.25	0.0134112	10,691	0.10 pp. (−0.04 pp., 0.23 pp)	0.000976267	0.0006898	0.157	1.000	NA	NA
0–4	Whole year	Outbreak	5.16%	Turbidity	50p = 0.13, 75p = 0.18, 95p = 0.34	0.0134238	10,691	−0.72 pp. (−2.93 pp., 1.50 pp)	−0.00715791	0.0113109	0.527	1.000	NA	NA
0–4	Winter	Outbreak	5.79%	Colour	50p = 5.75, 75p = 8.60, 95p = 14.25	0.0071784	2333	−0.24 pp. (−0.51 pp., 0.04 pp)	−0.00236996	0.0014064	0.092	1.000	NA	NA
0–4	Winter	Outbreak	5.81%	Turbidity	50p = 0.13, 75p = 0.19, 95p = 0.39	0.0076567	2341	−4.56 pp. (−9.07 pp., −0.06 pp)	−0.04563739	0.0229782	0.047	1.000	NA	NA
0–4	Spring	Outbreak	7.31%	Colour	50p = 5.50, 75p = 7.82, 95p = 14.50	0.0285614	2723	0.88 pp. (0.51 pp., 1.24 pp)	0.008760501	0.0018779	<0.001	<0.001*	NA	NA
0–4	Spring	Outbreak	7.31%	Turbidity	50p = 0.13, 75p = 0.19, 95p = 0.34	0.0153417	2723	2.65 pp. (−2.25 pp., 7.54 pp)	0.026458356	0.0249797	0.290	1.000	NA	NA
0–4	Summer	Outbreak	3.33%	Colour	50p = 5.00, 75p = 7.72, 95p = 14.00	0.0070623	2791	0.05 pp. (−0.16 pp., 0.26 pp)	0.000492113	0.0010614	0.643	1.000	NA	NA
0–4	Summer	Outbreak	3.27%	Turbidity	50p = 0.13, 75p = 0.17, 95p = 0.31	0.0073228	2782	−0.13 pp. (−2.98 pp., 2.71 pp)	−0.00133824	0.0145329	0.927	1.000	NA	NA
0–4	Autumn	Outbreak	4.40%	Colour	50p = 6.12, 75p = 8.45, 95p = 14.00	0.0026847	2844	0.03 pp. (−0.22 pp., 0.28 pp)	0.00027102	0.001284	0.833	1.000	NA	NA
0–4	Autumn	Outbreak	4.43%	Turbidity	50p = 0.12, 75p = 0.17, 95p = 0.31	0.0026161	2845	−1.67 pp. (−11.16 pp., 7.82 pp)	−0.01670648	0.0484341	0.730	1.000	NA	NA

**Table B.2:** Raw water and outbreaks.

Age	Season	Outcome	Outcome Summary	Exposure	Exposure Summary	Marginal_r2	n	Effect	Coef	Coef_se	Pval	PvalBonf	Interaction PvalAge	Interaction PvalSeason
Totalt	Whole year	Outbreak	6.18%	Coliform bacteria	50p = 2.62, 75p = 8.50, 95p = 74.88	0.0244364	10,263	−0.00 pp. (−0.01 pp., 0.01 pp)	−3.48E−05	5.875E-05	0.554	1.000	<0.001	<0.001
Totalt	Whole year	Outbreak	6.05%	Colour	50p = 24.35, 75p = 43.75, 95p = 75.38	0.0297182	9596	-0.02 pp. (−0.04 pp., 0.01 pp)	−0.000166	0.0001225	0.175	1.000	<0.001	<0.001
Totalt	Whole year	Outbreak	6.35%	*E. Coli*	50p = 0.75, 75p = 8.05, 95p = 26.92	0.0276459	9013	0.03 pp. (−0.04 pp., 0.10 pp)	0.000326	0.0003644	0.371	1.000	<0.001	<0.001
Totalt	Whole year	Outbreak	5.71%	Intestinal Enterococci	50p = 0.00, 75p = 0.25, 95p = 3.00	0.0244258	7705	−0.03 pp. (−0.26 pp., 0.20 pp)	−0.000265	0.0011788	0.822	1.000	<0.001	<0.001
Totalt	Whole year	Outbreak	6.10%	Turbidity	50p = 0.37, 75p = 1.75, 95p = 11.25	0.0292948	10,104	−0.08 pp. (−0.19 pp., 0.04 pp)	−0.000776	0.0005754	0.177	1.000	<0.001	<0.001
Totalt	Winter	Outbreak	6.99%	Coliform bacteria	50p = 2.88, 75p = 10.00, 95p = 61.00	0.0030813	2247	0.00 pp. (−0.04 pp., 0.04 pp)	2.59E−05	0.0002162	0.905	1.000	NA	NA
Totalt	Winter	Outbreak	6.79%	Colour	50p = 25.81, 75p = 47.75, 95p = 85.63	0.0040381	2120	-0.02 pp. (−0.06 pp., 0.03 pp)	−0.000158	0.0002178	0.468	1.000	NA	NA
Totalt	Winter	Outbreak	7.12%	*E. Coli*	50p = 0.88, 75p = 7.57, 95p = 25.65	0.0074697	1979	0.23 pp. (0.06 pp., 0.40 pp)	0.002309	0.0008471	0.006	0.801	NA	NA
Totalt	Winter	Outbreak	6.40%	Intestinal Enterococci	50p = 0.00, 75p = 0.50, 95p = 6.00	0.002112	1719	−0.08 pp. (−0.48 pp., 0.33 pp)	−0.000784	0.0020705	0.705	1.000	NA	NA
Totalt	Winter	Outbreak	6.93%	Turbidity	50p = 0.36, 75p = 1.93, 95p = 15.25	0.0027568	2238	0.03 pp. (−0.20 pp., 0.27 pp)	0.000322	0.0012011	0.789	1.000	NA	NA
Totalt	Spring	Outbreak	5.88%	Coliform bacteria	50p = 2.00, 75p = 4.04, 95p = 17.75	0.0241662	2604	0.03 pp. (−0.05 pp., 0.11 pp)	0.00027	0.000399	0.498	1.000	NA	NA
Totalt	Spring	Outbreak	5.55%	Colour	50p = 24.25, 75p = 46.00, 95p = 79.75	0.0206768	2433	−0.04 pp. (−0.08 pp., 0.00 pp)	−0.000369	0.0002045	0.071	1.000	NA	NA
Totalt	Spring	Outbreak	5.72%	*E. Coli*	50p = 0.25, 75p = 7.92, 95p = 27.18	0.0226513	2292	−0.09 pp. (−0.21 pp., 0.03 pp)	−0.000899	0.0006186	0.146	1.000	NA	NA
Totalt	Spring	Outbreak	5.10%	Intestinal Enterococci	50p = 0.00, 75p = 0.12, 95p = 1.75	0.0191172	1920	−0.36 pp. (−1.00 pp., 0.29 pp)	−0.003551	0.0032929	0.281	1.000	NA	NA
Totalt	Spring	Outbreak	5.77%	Turbidity	50p = 0.33, 75p = 1.81, 95p = 13.24	0.0228361	2567	−0.11 pp. (−0.29 pp., 0.08 pp)	−0.001073	0.0009355	0.251	1.000	NA	NA
Totalt	Summer	Outbreak	8.61%	Coliform bacteria	50p = 2.16, 75p = 6.30, 95p = 66.75	0.0315913	2671	−0.00 pp. (−0.04 pp., 0.03 pp)	−1.45E−05	0.0001834	0.937	1.000	NA	NA
Totalt	Summer	Outbreak	9.16%	Colour	50p = 23.41, 75p = 40.00, 95p = 65.50	0.0383202	2490	0.00 pp. (−0.07 pp., 0.08 pp)	4.19E-05	0.0004027	0.917	1.000	NA	NA
Totalt	Summer	Outbreak	9.37%	*E. Coli*	50p = 0.38, 75p = 8.05, 95p = 27.46	0.0357697	2338	0.08 pp. (−0.09 pp., 0.26 pp)	0.000822	0.0008835	0.352	1.000	NA	NA
Totalt	Summer	Outbreak	8.28%	Intestinal Enterococci	50p = 0.00, 75p = 0.10, 95p = 1.00	0.0353582	2004	0.91 pp. (−0.70 pp., 2.51 pp)	0.009071	0.0081886	0.268	1.000	NA	NA
Totalt	Summer	Outbreak	9.07%	Turbidity	50p = 0.36, 75p = 1.56, 95p = 8.69	0.0369385	2623	-0.24 pp. (−0.62 pp., 0.14 pp)	−0.00239	0.001931	0.216	1.000	NA	NA
Totalt	Autumn	Outbreak	3.43%	Coliform bacteria	50p = 4.12, 75p = 25.12, 95p = 155.75	0.0021585	2741	−0.01 pp. (−0.02 pp., 0.00 pp)	−5.41E−05	5.189E-05	0.297	1.000	NA	NA
Totalt	Autumn	Outbreak	2.90%	Colour	50p = 24.50, 75p = 41.37, 95p = 71.75	0.0010444	2553	0.00 pp. (−0.03 pp., 0.04 pp)	1.79E-05	0.0001697	0.916	1.000	NA	NA
Totalt	Autumn	Outbreak	3.37%	*E. Coli*	50p = 2.00, 75p = 8.50, 95p = 26.16	0.0022948	2404	-0.03 pp. (−0.12 pp., 0.06 pp)	−0.000289	0.0004642	0.534	1.000	NA	NA
Totalt	Autumn	Outbreak	3.20%	Intestinal Enterococci	50p = 0.00, 75p = 0.38, 95p = 5.50	0.0022456	2062	0.01 pp. (−0.25 pp., 0.26 pp)	7.06E−05	0.0012924	0.956	1.000	NA	NA
Totalt	Autumn	Outbreak	2.80%	Turbidity	50p = 0.44, 75p = 1.62, 95p = 8.45	0.0017057	2676	-0.10 pp. (−0.28 pp., 0.08 pp)	−0.001012	0.0009043	0.263	1.000	NA	NA
65+	Whole year	Outbreak	5.02%	Coliform bacteria	50p = 2.62, 75p = 8.50, 95p = 74.88	0.00621	10,263	−0.01 pp. (−0.02 pp., 0.00 pp)	−5.72E−05	5.203E-05	0.272	1.000	NA	NA
65+	Whole year	Outbreak	4.87%	Colour	50p = 24.35, 75p = 43.75, 95p = 75.38	0.0080371	9596	-0.01 pp. (−0.03 pp., 0.01 pp)	−6.29E−05	0.0001003	0.530	1.000	NA	NA
65+	Whole year	Outbreak	5.03%	*E. Coli*	50p = 0.75, 75p = 8.05, 95p = 26.92	0.0074005	9013	0.03 pp. (−0.03 pp., 0.09 pp)	0.000273	0.0003031	0.367	1.000	NA	NA
65+	Whole year	Outbreak	4.83%	Intestinal Enterococci	50p = 0.00, 75p = 0.25, 95p = 3.00	0.0074246	7705	0.07 pp. (−0.14 pp., 0.28 pp)	0.000679	0.0010705	0.526	1.000	NA	NA
65+	Whole year	Outbreak	4.88%	Turbidity	50p = 0.37, 75p = 1.75, 95p = 11.25	0.0070998	10,104	0.01 pp. (−0.09 pp., 0.11 pp)	0.000114	0.0004992	0.819	1.000	NA	NA
65+	Winter	Outbreak	5.21%	Coliform bacteria	50p = 2.88, 75p = 10.00, 95p = 61.00	0.0002085	2247	0.00 pp. (−0.03 pp., 0.04 pp)	2.37E-05	0.0001796	0.895	1.000	NA	NA
65+	Winter	Outbreak	5.09%	Colour	50p = 25.81, 75p = 47.75, 95p = 85.63	7.698E-05	2120	-0.00 pp. (−0.04 pp., 0.04 pp)	−3.16E−05	0.0002007	0.875	1.000	NA	NA
65+	Winter	Outbreak	5.31%	*E. Coli*	50p = 0.88, 75p = 7.57, 95p = 25.65	0.0005077	1979	0.06 pp. (−0.08 pp., 0.20 pp)	0.000576	0.0007077	0.415	1.000	NA	NA
65+	Winter	Outbreak	5.06%	Intestinal Enterococci	50p = 0.00, 75p = 0.50, 95p = 6.00	0.0005516	1719	0.01 pp. (−0.35 pp., 0.38 pp)	0.000138	0.0018665	0.941	1.000	NA	NA
65+	Winter	Outbreak	5.05%	Turbidity	50p = 0.36, 75p = 1.93, 95p = 15.25	0.0002647	2238	-0.06 pp. (−0.24 pp., 0.13 pp)	−0.000574	0.00095	0.546	1.000	NA	NA
65+	Spring	Outbreak	4.61%	Coliform bacteria	50p = 2.00, 75p = 4.04, 95p = 17.75	0.0076567	2604	−0.00 pp. (−0.07 pp., 0.07 pp)	−1.25E−05	0.0003587	0.972	1.000	NA	NA
65+	Spring	Outbreak	4.40%	Colour	50p = 24.25, 75p = 46.00, 95p = 79.75	0.0087268	2433	-0.00 pp. (−0.04 pp., 0.03 pp)	−2.98E−05	0.00018	0.868	1.000	NA	NA
65+	Spring	Outbreak	4.54%	*E. Coli*	50p = 0.25, 75p = 7.92, 95p = 27.18	0.0069057	2292	0.02 pp. (−0.09 pp., 0.13 pp)	0.000203	0.0005693	0.721	1.000	NA	NA
65+	Spring	Outbreak	4.22%	Intestinal Enterococci	50p = 0.00, 75p = 0.12, 95p = 1.75	0.0055532	1920	-0.03 pp. (−0.61 pp., 0.56 pp)	−0.000251	0.0029866	0.933	1.000	NA	NA
65+	Spring	Outbreak	4.44%	Turbidity	50p = 0.33, 75p = 1.81, 95p = 13.24	0.0089858	2567	0.06 pp. (−0.10 pp., 0.22 pp)	0.000582	0.0008326	0.484	1.000	NA	NA
65+	Summer	Outbreak	6.36%	Coliform bacteria	50p = 2.16, 75p = 6.30, 95p = 66.75	0.0086467	2671	−0.03 pp. (−0.06 pp., 0.01 pp)	−0.000262	0.0001632	0.108	1.000	NA	NA
65+	Summer	Outbreak	6.47%	Colour	50p = 23.41, 75p = 40.00, 95p = 65.50	0.0098172	2490	−0.02 pp. (−0.09 pp., 0.04 pp)	−0.000205	0.0003298	0.533	1.000	NA	NA
65+	Summer	Outbreak	6.67%	*E. Coli*	50p = 0.38, 75p = 8.05, 95p = 27.46	0.0090047	2338	0.06 pp. (−0.09 pp., 0.21 pp)	0.000612	0.000755	0.418	1.000	NA	NA
65+	Summer	Outbreak	6.44%	Intestinal Enterococci	50p = 0.00, 75p = 0.10, 95p = 1.00	0.0104178	2004	−0.45 pp. (−1.90 pp., 0.99 pp)	−0.00452	0.0073641	0.539	1.000	NA	NA
65+	Summer	Outbreak	6.37%	Turbidity	50p = 0.36, 75p = 1.56, 95p = 8.69	0.0080785	2623	−0.02 pp. (−0.36 pp., 0.31 pp)	−0.000228	0.0017155	0.894	1.000	NA	NA
65+	Autumn	Outbreak	3.94%	Coliform bacteria	50p = 4.12, 75p = 25.12, 95p = 155.75	0.0003423	2741	−0.00 pp. (−0.01 pp., 0.01 pp)	−2.11E−05	6.024E-05	0.726	1.000	NA	NA
65+	Autumn	Outbreak	3.56%	Colour	50p = 24.50, 75p = 41.37, 95p = 71.75	0.0008603	2553	0.00 pp. (−0.04 pp., 0.04 pp)	1.13E-05	0.0002086	0.957	1.000	NA	NA
65+	Autumn	Outbreak	3.66%	*E. Coli*	50p = 2.00, 75p = 8.50, 95p = 26.16	0.0005489	2404	0.00 pp. (−0.10 pp., 0.11 pp)	3.62E-05	0.0005208	0.945	1.000	NA	NA
65+	Autumn	Outbreak	3.64%	Intestinal Enterococci	50p = 0.00, 75p = 0.38, 95p = 5.50	0.0006178	2062	0.12 pp. (−0.15 pp., 0.40 pp)	0.001231	0.0014097	0.383	1.000	NA	NA
65+	Autumn	Outbreak	3.70%	Turbidity	50p = 0.44, 75p = 1.62, 95p = 8.45	0.0018628	2676	0.16 pp. (−0.07 pp., 0.38 pp)	0.001596	0.0011478	0.164	1.000	NA	NA
5–14	Whole year	Outbreak	5.48%	Coliform bacteria	50p = 2.62, 75p = 8.50, 95p = 74.88	0.0070554	10,263	-0.00 pp. (−0.01 pp., 0.01 pp)	−3.69E−05	5.42E-05	0.496	1.000	NA	NA
5–14	Whole year	Outbreak	5.36%	Colour	50p = 24.35, 75p = 43.75, 95p = 75.38	0.0092029	9596	-0.02 pp. (−0.04 pp., 0.00 pp)	−0.000187	0.0001049	0.075	1.000	NA	NA
5–14	Whole year	Outbreak	5.41%	*E. Coli*	50p = 0.75, 75p = 8.05, 95p = 26.92	0.0082571	9013	−0.02 pp. (−0.08 pp., 0.03 pp)	−0.000247	0.0003039	0.417	1.000	NA	NA
5–14	Whole year	Outbreak	5.43%	Intestinal Enterococci	50p = 0.00, 75p = 0.25, 95p = 3.00	0.0085881	7705	−0.29 pp. (−0.51 pp., −0.07 pp)	−0.002874	0.0011181	0.010	1.000	NA	NA
5–14	Whole year	Outbreak	5.31%	Turbidity	50p = 0.37, 75p = 1.75, 95p = 11.25	0.0086039	10,104	−0.15 pp. (−0.25 pp., −0.05 pp)	−0.001506	0.0005195	0.004	0.467	NA	NA
5–14	Winter	Outbreak	4.36%	Coliform bacteria	50p = 2.88, 75p = 10.00, 95p = 61.00	0.0008938	2247	−0.02 pp. (−0.05 pp., 0.01 pp)	−0.000222	0.0001637	0.175	1.000	NA	NA
5–14	Winter	Outbreak	4.39%	Colour	50p = 25.81, 75p = 47.75, 95p = 85.63	0.0016481	2120	−0.02 pp. (−0.06 pp., 0.01 pp)	−0.000246	0.0001717	0.152	1.000	NA	NA
5–14	Winter	Outbreak	4.45%	*E. Coli*	50p = 0.88, 75p = 7.57, 95p = 25.65	0.0015362	1979	0.06 pp. (−0.06 pp., 0.19 pp)	0.000642	0.0006437	0.318	1.000	NA	NA
5–14	Winter	Outbreak	5.06%	Intestinal Enterococci	50p = 0.00, 75p = 0.50, 95p = 6.00	0.0048919	1719	−0.31 pp. (−0.67 pp., 0.04 pp)	−0.003137	0.0018185	0.085	1.000	NA	NA
5–14	Winter	Outbreak	4.20%	Turbidity	50p = 0.36, 75p = 1.93, 95p = 15.25	0.0014343	2238	−0.14 pp. (−0.30 pp., 0.02 pp)	−0.001412	0.0008124	0.082	1.000	NA	NA
5–14	Spring	Outbreak	5.88%	Coliform bacteria	50p = 2.00, 75p = 4.04, 95p = 17.75	0.0061218	2604	0.01 pp. (−0.08 pp., 0.09 pp)	6.16E−05	0.0004185	0.883	1.000	NA	NA
5–14	Spring	Outbreak	6.04%	Colour	50p = 24.25, 75p = 46.00, 95p = 79.75	0.0098692	2433	-0.04 pp. (−0.09 pp., 0.01 pp)	−0.0004	0.0002408	0.097	1.000	NA	NA
5–14	Spring	Outbreak	5.80%	*E. Coli*	50p = 0.25, 75p = 7.92, 95p = 27.18	0.0089071	2292	−0.12 pp. (−0.26 pp., 0.01 pp)	−0.001237	0.0006949	0.075	1.000	NA	NA
5–14	Spring	Outbreak	5.57%	Intestinal Enterococci	50p = 0.00, 75p = 0.12, 95p = 1.75	0.0092983	1920	−0.71 pp. (−1.40 pp., −0.01 pp)	−0.007054	0.0035406	0.046	1.000	NA	NA
5–14	Spring	Outbreak	5.88%	Turbidity	50p = 0.33, 75p = 1.81, 95p = 13.24	0.0093343	2567	−0.28 pp. (−0.48 pp., −0.08 pp)	−0.002778	0.0010122	0.006	0.757	NA	NA
5–14	Summer	Outbreak	7.11%	Coliform bacteria	50p = 2.16, 75p = 6.30, 95p = 66.75	0.0079615	2671	−0.01 pp. (−0.04 pp., 0.03 pp)	−5.9E-05	0.0001626	0.717	1.000	NA	NA
5–14	Summer	Outbreak	6.91%	Colour	50p = 23.41, 75p = 40.00, 95p = 65.50	0.01056	2490	0.01 pp. (−0.04 pp., 0.07 pp)	0.000149	0.0002819	0.598	1.000	NA	NA
5–14	Summer	Outbreak	7.31%	*E. Coli*	50p = 0.38, 75p = 8.05, 95p = 27.46	0.0074555	2338	0.01 pp. (−0.12 pp., 0.15 pp)	0.00013	0.0006757	0.848	1.000	NA	NA
5–14	Summer	Outbreak	6.89%	Intestinal Enterococci	50p = 0.00, 75p = 0.10, 95p = 1.00	0.0109902	2004	−1.12 pp. (−2.59 pp., 0.35 pp)	−0.011194	0.0074883	0.135	1.000	NA	NA
5–14	Summer	Outbreak	6.98%	Turbidity	50p = 0.36, 75p = 1.56, 95p = 8.69	0.0090673	2623	−0.02 pp. (−0.31 pp., 0.27 pp)	−0.000174	0.0014712	0.906	1.000	NA	NA
5–14	Autumn	Outbreak	4.41%	Coliform bacteria	50p = 4.12, 75p = 25.12, 95p = 155.75	0.0007733	2741	−0.00 pp. (−0.01 pp., 0.01 pp)	−1.27E−05	6.156E-05	0.837	1.000	NA	NA
5–14	Autumn	Outbreak	4.00%	Colour	50p = 24.50, 75p = 41.37, 95p = 71.75	0.000607	2553	-0.02 pp. (−0.05 pp., 0.02 pp)	−0.000172	0.0001907	0.367	1.000	NA	NA
5–14	Autumn	Outbreak	3.99%	*E. Coli*	50p = 2.00, 75p = 8.50, 95p = 26.16	0.0006539	2404	−0.02 pp. (−0.12 pp., 0.08 pp)	−0.000195	0.0005151	0.705	1.000	NA	NA
5–14	Autumn	Outbreak	4.17%	Intestinal Enterococci	50p = 0.00, 75p = 0.38, 95p = 5.50	0.0007518	2062	−0.13 pp. (−0.42 pp., 0.16 pp)	−0.001313	0.0014794	0.375	1.000	NA	NA
5–14	Autumn	Outbreak	4.07%	Turbidity	50p = 0.44, 75p = 1.62, 95p = 8.45	0.0006017	2676	−0.07 pp. (−0.28 pp., 0.14 pp)	−0.000709	0.0010786	0.511	1.000	NA	NA
15–64	Whole year	Outbreak	5.98%	Coliform bacteria	50p = 2.62, 75p = 8.50, 95p = 74.88	0.0293571	10,263	−0.01 pp. (−0.02 pp., 0.01 pp)	−5.09E−05	5.842E-05	0.383	1.000	NA	NA
15–64	Whole year	Outbreak	5.81%	Colour	50p = 24.35, 75p = 43.75, 95p = 75.38	0.0351078	9596	-0.01 pp. (−0.04 pp., 0.01 pp)	−0.000101	0.0001279	0.428	1.000	NA	NA
15–64	Whole year	Outbreak	6.15%	*E. Coli*	50p = 0.75, 75p = 8.05, 95p = 26.92	0.0335022	9013	0.01 pp. (−0.06 pp., 0.08 pp)	9.77E−05	0.0003388	0.773	1.000	NA	NA
15–64	Whole year	Outbreak	5.74%	Intestinal Enterococci	50p = 0.00, 75p = 0.25, 95p = 3.00	0.0326902	7705	-0.05 pp. (−0.29 pp., 0.18 pp)	−0.000523	0.0011982	0.662	1.000	NA	NA
15–64	Whole year	Outbreak	5.79%	Turbidity	50p = 0.37, 75p = 1.75, 95p = 11.25	0.0334184	10,104	−0.04 pp. (−0.16 pp., 0.08 pp)	−0.000366	0.0006182	0.554	1.000	NA	NA
15–64	Winter	Outbreak	7.57%	Coliform bacteria	50p = 2.88, 75p = 10.00, 95p = 61.00	0.0014715	2247	−0.02 pp. (−0.06 pp., 0.02 pp)	−0.000188	0.0002183	0.389	1.000	NA	NA
15–64	Winter	Outbreak	7.31%	Colour	50p = 25.81, 75p = 47.75, 95p = 85.63	0.0028285	2120	−0.00 pp. (−0.05 pp., 0.04 pp)	−1.07E−05	0.0002335	0.963	1.000	NA	NA
15–64	Winter	Outbreak	7.78%	*E. Coli*	50p = 0.88, 75p = 7.57, 95p = 25.65	0.0012696	1979	0.04 pp. (−0.13 pp., 0.21 pp)	0.000421	0.0008559	0.623	1.000	NA	NA
15–64	Winter	Outbreak	7.21%	Intestinal Enterococci	50p = 0.00, 75p = 0.50, 95p = 6.00	0.001752	1719	-0.23 pp. (−0.68 pp., 0.21 pp)	−0.002317	0.0022683	0.307	1.000	NA	NA
15–64	Winter	Outbreak	7.15%	Turbidity	50p = 0.36, 75p = 1.93, 95p = 15.25	0.0023638	2238	−0.06 pp. (−0.28 pp., 0.16 pp)	−0.000617	0.0011198	0.582	1.000	NA	NA
15–64	Spring	Outbreak	3.46%	Coliform bacteria	50p = 2.00, 75p = 4.04, 95p = 17.75	0.0078247	2604	0.02 pp. (−0.05 pp., 0.08 pp)	0.000175	0.0003211	0.586	1.000	NA	NA
15–64	Spring	Outbreak	3.16%	Colour	50p = 24.25, 75p = 46.00, 95p = 79.75	0.0077253	2433	−0.05 pp. (−0.08 pp., −0.01 pp)	−0.000478	0.0001753	0.006	0.797	NA	NA
15–64	Spring	Outbreak	3.40%	*E. Coli*	50p = 0.25, 75p = 7.92, 95p = 27.18	0.009227	2292	−0.01 pp. (−0.12 pp., 0.10 pp)	−9.17E−05	0.0005436	0.866	1.000	NA	NA
15–64	Spring	Outbreak	3.18%	Intestinal Enterococci	50p = 0.00, 75p = 0.12, 95p = 1.75	0.002258	1920	-0.15 pp. (−0.69 pp., 0.39 pp)	−0.001491	0.0027593	0.589	1.000	NA	NA
15–64	Spring	Outbreak	3.27%	Turbidity	50p = 0.33, 75p = 1.81, 95p = 13.24	0.0057587	2567	−0.11 pp. (−0.28 pp., 0.05 pp)	−0.001123	0.0008304	0.176	1.000	NA	NA
15–64	Summer	Outbreak	10.07%	Coliform bacteria	50p = 2.16, 75p = 6.30, 95p = 66.75	0.029251	2671	0.02 pp. (−0.02 pp., 0.06 pp)	0.000178	0.0002037	0.382	1.000	NA	NA
15–64	Summer	Outbreak	10.24%	Colour	50p = 23.41, 75p = 40.00, 95p = 65.50	0.0365882	2490	0.05 pp. (−0.04 pp., 0.15 pp)	0.000532	0.0004996	0.287	1.000	NA	NA
15–64	Summer	Outbreak	10.56%	*E. Coli*	50p = 0.38, 75p = 8.05, 95p = 27.46	0.0338023	2338	0.02 pp. (−0.17 pp., 0.21 pp)	0.000195	0.0009874	0.843	1.000	NA	NA
15–64	Summer	Outbreak	9.83%	Intestinal Enterococci	50p = 0.00, 75p = 0.10, 95p = 1.00	0.0373596	2004	0.43 pp. (−1.32 pp., 2.17 pp)	0.004272	0.0089068	0.632	1.000	NA	NA
15–64	Summer	Outbreak	10.18%	Turbidity	50p = 0.36, 75p = 1.56, 95p = 8.69	0.0341717	2623	0.28 pp. (−0.19 pp., 0.75 pp)	0.00282	0.0023869	0.237	1.000	NA	NA
15–64	Autumn	Outbreak	3.10%	Coliform bacteria	50p = 4.12, 75p = 25.12, 95p = 155.75	0.0015984	2741	−0.01 pp. (−0.02 pp., 0.00 pp)	−7.61E−05	4.782E-05	0.111	1.000	NA	NA
15–64	Autumn	Outbreak	2.78%	Colour	50p = 24.50, 75p = 41.37, 95p = 71.75	0.0012194	2553	-0.01 pp. (−0.05 pp., 0.02 pp)	−0.000135	0.0001719	0.433	1.000	NA	NA
15–64	Autumn	Outbreak	3.12%	*E. Coli*	50p = 2.00, 75p = 8.50, 95p = 26.16	0.0019263	2404	0.06 pp. (−0.02 pp., 0.15 pp)	0.000634	0.0004313	0.142	1.000	NA	NA
15–64	Autumn	Outbreak	2.91%	Intestinal Enterococci	50p = 0.00, 75p = 0.38, 95p = 5.50	0.0012055	2062	0.03 pp. (−0.20 pp., 0.26 pp)	0.000319	0.0011736	0.786	1.000	NA	NA
15–64	Autumn	Outbreak	2.77%	Turbidity	50p = 0.44, 75p = 1.62, 95p = 8.45	0.0011504	2676	−0.03 pp. (−0.21 pp., 0.16 pp)	−0.000262	0.0009429	0.781	1.000	NA	NA
0–4	Whole year	Outbreak	5.33%	Coliform bacteria	50p = 2.62, 75p = 8.50, 95p = 74.88	0.0167972	10,263	−0.00 pp. (−0.02 pp., 0.01 pp)	−4.91E−05	5.438E-05	0.367	1.000	NA	NA
0–4	Whole year	Outbreak	5.29%	Colour	50p = 24.35, 75p = 43.75, 95p = 75.38	0.0157644	9596	0.00 pp. (−0.02 pp., 0.02 pp)	1.91E-05	0.000104	0.855	1.000	NA	NA
0–4	Whole year	Outbreak	5.26%	*E. Coli*	50p = 0.75, 75p = 8.05, 95p = 26.92	0.0176369	9013	0.00 pp. (−0.06 pp., 0.06 pp)	7.29E-06	0.0002984	0.981	1.000	NA	NA
0–4	Whole year	Outbreak	5.13%	Intestinal Enterococci	50p = 0.00, 75p = 0.25, 95p = 3.00	0.014943	7705	-0.06 pp. (−0.27 pp., 0.16 pp)	−0.000551	0.0010851	0.612	1.000	NA	NA
0–4	Whole year	Outbreak	5.35%	Turbidity	50p = 0.37, 75p = 1.75, 95p = 11.25	0.0171297	10,104	−0.04 pp. (−0.14 pp., 0.07 pp)	−0.00037	0.0005432	0.495	1.000	NA	NA
0–4	Winter	Outbreak	5.21%	Coliform bacteria	50p = 2.88, 75p = 10.00, 95p = 61.00	0.0041164	2247	0.00 pp. (−0.04 pp., 0.04 pp)	4.31E−06	0.0001856	0.981	1.000	NA	NA
0–4	Winter	Outbreak	5.47%	Colour	50p = 25.81, 75p = 47.75, 95p = 85.63	0.0046307	2120	-0.01 pp. (−0.05 pp., 0.03 pp)	−0.000115	0.0001942	0.553	1.000	NA	NA
0–4	Winter	Outbreak	5.36%	*E. Coli*	50p = 0.88, 75p = 7.57, 95p = 25.65	0.0044627	1979	0.13 pp. (−0.02 pp., 0.28 pp)	0.001273	0.000761	0.094	1.000	NA	NA
0–4	Winter	Outbreak	5.29%	Intestinal Enterococci	50p = 0.00, 75p = 0.50, 95p = 6.00	0.000592	1719	−0.05 pp. (−0.42 pp., 0.32 pp)	−0.000505	0.0018858	0.789	1.000	NA	NA
0–4	Winter	Outbreak	5.63%	Turbidity	50p = 0.36, 75p = 1.93, 95p = 15.25	0.0050625	2238	0.02 pp. (−0.18 pp., 0.21 pp)	0.000163	0.0010026	0.870	1.000	NA	NA
0–4	Spring	Outbreak	8.10%	Coliform bacteria	50p = 2.00, 75p = 4.04, 95p = 17.75	0.0226149	2604	−0.07 pp. (−0.17 pp., 0.02 pp)	−0.000729	0.0004935	0.139	1.000	NA	NA
0–4	Spring	Outbreak	7.89%	Colour	50p = 24.25, 75p = 46.00, 95p = 79.75	0.0194404	2433	0.00 pp. (−0.06 pp., 0.06 pp)	9.06E−06	0.0002979	0.976	1.000	NA	NA
0–4	Spring	Outbreak	8.07%	*E. Coli*	50p = 0.25, 75p = 7.92, 95p = 27.18	0.0243252	2292	-0.15 pp. (−0.30 pp., 0.01 pp)	−0.001475	0.0007798	0.058	1.000	NA	NA
0–4	Spring	Outbreak	8.02%	Intestinal Enterococci	50p = 0.00, 75p = 0.12, 95p = 1.75	0.018113	1920	−0.80 pp. (−1.61 pp., 0.00 pp)	−0.008047	0.0041097	0.050	1.000	NA	NA
0–4	Spring	Outbreak	8.14%	Turbidity	50p = 0.33, 75p = 1.81, 95p = 13.24	0.0244176	2567	−0.35 pp. (−0.65 pp., −0.05 pp)	−0.003543	0.0015293	0.021	1.000	NA	NA
0–4	Summer	Outbreak	3.48%	Coliform bacteria	50p = 2.16, 75p = 6.30, 95p = 66.75	0.0043619	2671	0.00 pp. (−0.02 pp., 0.03 pp)	3.85E−05	0.0001182	0.745	1.000	NA	NA
0–4	Summer	Outbreak	3.57%	Colour	50p = 23.41, 75p = 40.00, 95p = 65.50	0.0081968	2490	0.01 pp. (−0.04 pp., 0.05 pp)	7.12E-05	0.0002377	0.765	1.000	NA	NA
0–4	Summer	Outbreak	3.29%	*E. Coli*	50p = 0.38, 75p = 8.05, 95p = 27.46	0.0043511	2338	0.03 pp. (−0.06 pp., 0.13 pp)	0.000344	0.0004844	0.478	1.000	NA	NA
0–4	Summer	Outbreak	3.14%	Intestinal Enterococci	50p = 0.00, 75p = 0.10, 95p = 1.00	0.0048294	2004	-0.29 pp. (−1.33 pp., 0.74 pp)	−0.002944	0.0052594	0.576	1.000	NA	NA
0–4	Summer	Outbreak	3.77%	Turbidity	50p = 0.36, 75p = 1.56, 95p = 8.69	0.0075229	2623	0.12 pp. (−0.11 pp., 0.35 pp)	0.001223	0.001171	0.296	1.000	NA	NA
0–4	Autumn	Outbreak	4.60%	Coliform bacteria	50p = 4.12, 75p = 25.12, 95p = 155.75	0.0045285	2741	−0.00 pp. (−0.02 pp., 0.01 pp)	−3.84E−05	6.48E−05	0.554	1.000	NA	NA
0–4	Autumn	Outbreak	4.35%	Colour	50p = 24.50, 75p = 41.37, 95p = 71.75	0.0034002	2553	0.02 pp. (−0.03 pp., 0.07 pp)	0.000221	0.0002426	0.362	1.000	NA	NA
0–4	Autumn	Outbreak	4.41%	*E. Coli*	50p = 2.00, 75p = 8.50, 95p = 26.16	0.0060558	2404	0.01 pp. (−0.09 pp., 0.12 pp)	0.000141	0.0005552	0.799	1.000	NA	NA
0–4	Autumn	Outbreak	4.22%	Intestinal Enterococci	50p = 0.00, 75p = 0.38, 95p = 5.50	0.0052037	2062	0.12 pp. (−0.16 pp., 0.40 pp)	0.001173	0.0014295	0.412	1.000	NA	NA
0–4	Autumn	Outbreak	4.00%	Turbidity	50p = 0.44, 75p = 1.62, 95p = 8.45	0.0029414	2676	−0.01 pp. (−0.25 pp., 0.23 pp)	-9E−05	0.0012179	0.941	1.000	NA	NA

**Table C:** Extreme weather and outbreaks.

Exposure	Age	Season	Effect	Pval	PvalBonf	Interaction PvalAge	Interaction PvalSeason
wp950_a_runoff0_3	Totalt	Whole year	−0.03 pp. (−0.06 pp., 0.00 pp)	0.098	1.000	0.001	0.007
wp950_a_runoff0_3	Totalt	Winter	−0.15 pp. (−0.22 pp., −0.07 pp)	<0.001	0.004*	NA	NA
wp950_a_runoff0_3	Totalt	Spring	−0.04 pp. (−0.09 pp., 0.01 pp)	0.130	1.000	NA	NA
wp950_a_runoff0_3	Totalt	Summer	0.04 pp. (−0.02 pp., 0.11 pp)	0.182	1.000	NA	NA
wp950_a_runoff0_3	Totalt	Autumn	0.04 pp. (−0.03 pp., 0.11 pp)	0.300	1.000	NA	NA
wp950_a_runoff0_3	65+	Whole year	−0.00 pp. (−0.03 pp., 0.03 pp)	0.983	1.000	NA	NA
wp950_a_runoff0_3	65+	Winter	−0.01 pp. (−0.08 pp., 0.06 pp)	0.791	1.000	NA	NA
wp950_a_runoff0_3	65+	Spring	−0.02 pp. (−0.07 pp., 0.02 pp)	0.359	1.000	NA	NA
wp950_a_runoff0_3	65+	Summer	0.02 pp. (−0.03 pp., 0.08 pp)	0.429	1.000	NA	NA
wp950_a_runoff0_3	65+	Autumn	0.02 pp. (−0.05 pp., 0.10 pp)	0.576	1.000	NA	NA
wp950_a_runoff0_3	15–64	Whole year	0.01 pp. (−0.02 pp., 0.04 pp)	0.556	1.000	NA	NA
wp950_a_runoff0_3	15–64	Winter	−0.11 pp. (−0.18 pp., −0.04 pp)	0.004	0.195	NA	NA
wp950_a_runoff0_3	15–64	Spring	0.04 pp. (−0.00 pp., 0.09 pp)	0.061	1.000	NA	NA
wp950_a_runoff0_3	15–64	Summer	0.05 pp. (−0.01 pp., 0.12 pp)	0.119	1.000	NA	NA
wp950_a_runoff0_3	15–64	Autumn	0.03 pp. (−0.04 pp., 0.10 pp)	0.434	1.000	NA	NA
wp950_a_runoff0_3	5–14	Whole year	−0.05 pp. (−0.08 pp., −0.02 pp)	<0.001	0.014*	NA	NA
wp950_a_runoff0_3	5–14	Winter	−0.08 pp. (−0.14 pp., −0.01 pp)	0.022	1.000	NA	NA
wp950_a_runoff0_3	5–14	Spring	−0.08 pp. (−0.12 pp., −0.03 pp)	0.001	0.061	NA	NA
wp950_a_runoff0_3	5–14	Summer	−0.02 pp. (−0.08 pp., 0.04 pp)	0.535	1.000	NA	NA
wp950_a_runoff0_3	5–14	Autumn	−0.03 pp. (−0.10 pp., 0.05 pp)	0.494	1.000	NA	NA
wp950_a_runoff0_3	0–4	Whole year	−0.02 pp. (−0.05 pp., 0.01 pp)	0.141	1.000	NA	NA
wp950_a_runoff0_3	0–4	Winter	−0.14 pp. (−0.21 pp., −0.07 pp)	<0.001	0.003*	NA	NA
wp950_a_runoff0_3	0–4	Spring	−0.06 pp. (−0.11 pp., −0.00 pp)	0.034	1.000	NA	NA
wp950_a_runoff0_3	0–4	Summer	0.03 pp. (−0.03 pp., 0.09 pp)	0.296	1.000	NA	NA
wp950_a_runoff0_3	0–4	Autumn	0.12 pp. (0.04 pp., 0.20 pp)	0.003	0.183	NA	NA
wp950_c_rain0_3	Totalt	Whole year	−0.21 pp. (−0.32 pp., −0.09 pp)	0.001	0.029*	<0.001	0.031
wp950_c_rain0_3	Totalt	Winter	−0.42 pp. (−0.72 pp., −0.13 pp)	0.005	0.255	NA	NA
wp950_c_rain0_3	Totalt	Spring	−0.30 pp. (−0.62 pp., 0.01 pp)	0.058	1.000	NA	NA
wp950_c_rain0_3	Totalt	Summer	−0.16 pp. (−0.40 pp., 0.08 pp)	0.187	1.000	NA	NA
wp950_c_rain0_3	Totalt	Autumn	−0.15 pp. (−0.32 pp., 0.02 pp)	0.082	1.000	NA	NA
wp950_c_rain0_3	65+	Whole year	0.02 pp. (−0.09 pp., 0.13 pp)	0.728	1.000	NA	NA
wp950_c_rain0_3	65+	Winter	−0.10 pp. (−0.37 pp., 0.18 pp)	0.485	1.000	NA	NA
wp950_c_rain0_3	65+	Spring	0.12 pp. (−0.18 pp., 0.41 pp)	0.443	1.000	NA	NA
wp950_c_rain0_3	65+	Summer	0.03 pp. (−0.19 pp., 0.24 pp)	0.812	1.000	NA	NA
wp950_c_rain0_3	65+	Autumn	0.02 pp. (−0.16 pp., 0.20 pp)	0.794	1.000	NA	NA
wp950_c_rain0_3	15–64	Whole year	−0.11 pp. (−0.23 pp., 0.01 pp)	0.067	1.000	NA	NA
wp950_c_rain0_3	15–64	Winter	−0.58 pp. (−0.88 pp., −0.29 pp)	<0.001	0.006*	NA	NA
wp950_c_rain0_3	15–64	Spring	0.37 pp. (0.09 pp., 0.65 pp)	0.010	0.531	NA	NA
wp950_c_rain0_3	15–64	Summer	−0.25 pp. (−0.51 pp., 0.01 pp)	0.059	1.000	NA	NA
wp950_c_rain0_3	15–64	Autumn	0.04 pp. (−0.13 pp., 0.21 pp)	0.664	1.000	NA	NA
wp950_c_rain0_3	5–14	Whole year	−0.06 pp. (−0.17 pp., 0.05 pp)	0.289	1.000	NA	NA
wp950_c_rain0_3	5–14	Winter	0.10 pp. (−0.16 pp., 0.36 pp)	0.454	1.000	NA	NA
wp950_c_rain0_3	5–14	Spring	−0.43 pp. (−0.73 pp., −0.13 pp)	0.005	0.274	NA	NA
wp950_c_rain0_3	5–14	Summer	0.05 pp. (−0.16 pp., 0.26 pp)	0.628	1.000	NA	NA
wp950_c_rain0_3	5–14	Autumn	−0.09 pp. (−0.27 pp., 0.09 pp)	0.316	1.000	NA	NA
wp950_c_rain0_3	0–4	Whole year	−0.09 pp. (−0.21 pp., 0.03 pp)	0.136	1.000	NA	NA
wp950_c_rain0_3	0–4	Winter	−0.12 pp. (−0.39 pp., 0.16 pp)	0.405	1.000	NA	NA
wp950_c_rain0_3	0–4	Spring	-0.75 pp. (−1.09 pp., −0.42 pp)	<0.001	0.001*	NA	NA
wp950_c_rain0_3	0–4	Summer	0.17 pp. (−0.02 pp., 0.37 pp)	0.082	1.000	NA	NA
wp950_c_rain0_3	0–4	Autumn	-0.05 pp. (−0.24 pp., 0.14 pp)	0.583	1.000	NA	NA
wp950_c_temperature0_3	Totalt	Whole year	0.11 pp. (0.08 pp., 0.15 pp)	<0.001	<0.001*	<0.001	<0.001
wp950_c_temperature0_3	65+	Whole year	0.04 pp. (0.01 pp., 0.07 pp)	0.013	0.716	NA	NA
wp950_c_temperature0_3	15–64	Whole year	0.18 pp. (0.14 pp., 0.21 pp)	<0.001	<0.001*	NA	NA
wp950_c_temperature0_3	5–14	Whole year	0.03 pp. (−0.00 pp., 0.06 pp)	0.075	1.000	NA	NA
wp950_c_temperature0_3	0–4	Whole year	-0.00 pp. (−0.04 pp., 0.03 pp)	0.767	1.000	NA	NA
